# Transendothelial Transport and Its Role in Therapeutics

**DOI:** 10.1155/2014/309404

**Published:** 2014-08-27

**Authors:** Ravi Kant Upadhyay

**Affiliations:** Department of Zoology, DDU Gorakhpur University, Gorakhpur 273009, UP, India

## Abstract

Present review paper highlights role of BBB in endothelial transport of various substances into the brain. More specifically, permeability functions of BBB in transendothelial transport of various substances such as metabolic fuels, ethanol, amino acids, proteins, peptides, lipids, vitamins, neurotransmitters, monocarbxylic acids, gases, water, and minerals in the peripheral circulation and into the brain have been widely explained. In addition, roles of various receptors, ATP powered pumps, channels, and transporters in transport of vital molecules in maintenance of homeostasis and normal body functions have been described in detail. Major role of integral membrane proteins, carriers, or transporters in drug transport is highlighted. Both diffusion and carrier mediated transport mechanisms which facilitate molecular trafficking through transcellular route to maintain influx and outflux of important nutrients and metabolic substances are elucidated. Present review paper aims to emphasize role of important transport systems with their recent advancements in CNS protection mainly for providing a rapid clinical aid to patients. This review also suggests requirement of new well-designed therapeutic strategies mainly potential techniques, appropriate drug formulations, and new transport systems for quick, easy, and safe delivery of drugs across blood brain barrier to save the life of tumor and virus infected patients.

## 1. Introduction

Blood brain barrier (BBB) is a vasculature of the central nervous system (CNS) that is formed by capillary endothelial cells. This is not a fixed structure but its function depends on the complex interplay between the different cell types such as the endothelial cells, astrocytes, pericytes, and the extracellular matrix of the brain and blood flow maintained in the microvessels or brain capillaries. BBB is physically located in endothelium of blood vessels (capillaries) and acts as a “physical barrier” due to formation of complex tight junctions between adjacent endothelial cells. Both luminal and abluminal membranes contain specific transport systems and regulate transcellular traffic. Thus, BBB facilitates most molecular traffic to transcellular route and maintain influx and outflux of important nutrients and metabolic substances. It acts as a rate limiting structure, which obstructs transcapillary movement of substrates in the peripheral circulation into the brain. This is a dynamic barrier which acts as a firewall of a computer, which permits transient flow of nutrients, gases, and smaller molecules into the brain and keeps out harmful metabolites such as drugs ions, toxins which potentially circulate in the blood stream. It also protects the brain from sudden rising blood pressure and transcapillary movement of substrates in the peripheral circulation into the brain. Thus, association of astrocytes with the brain endothelial cells provides a modular organization that allows precise control over the substances that enter or leave the brain.

The blood brain barrier actually consists of several components. It includes endothelial cells, astrocytes, and blood capillaries in the brain, which form the major structural component of the blood brain barrier. These are quite different from other capillaries found in the body as their endothelial wall possesses tight junctions which obstruct transport between cells. More often, only small molecules that can diffuse through these cells can cross the barrier. More specifically, the endothelial cells also possess transporters which show permeability characteristics and allow transport of oxygen and CO_2_ across the BBB, bu these selectively prevent other substances from crossing [1]. In addition, blood capillaries in the brain are enclosed by the flattened “end-feet” of astrocytic cells, which also act as a partial, active barrier. These are found in large numbers and maintain the functional integrity of BBB. These are highly specialized glial cells which have star like shape due to presence of long branches. These respond to CNS in a reactive process that is known as astrogliosis which is recognized as a pathological marker of neuropathologies and CNS disorders. These cells wrap themselves around the capillaries, like insulation on a wire, and display graded changes that result in specific signaling events. These cells show vast molecular arsenals at the disposal and show reactive astrogliosis and glial scar formation. There are two different types of astrocytes, that is, protoplasmic astrocytes and fibrous astrocytes. First type of astrocytes occurs in gray matter or specially areas rich in cell bodies, while second type occurs in white matter, that is, area formed mainly of axons. These cells also possess long dendrite like processes that overlap all the brain vessels and surround many synapses. These cells play primary role in synaptic transmission and information exchanges [2, 3] by operating through certain gradations, mechanisms, and transcellular functions both in healthy tissues [4, 5] and in the state of pathologies like ischemic injury [6–9]. Astrocytes influence polarity of blood brain barrier [10]. Transmitters and modulators released by neurons, astrocytes, and endothelium allow complex signaling between cells in the neurovascular unit, and many features of the BBB phenotype are subject to modulation under physiological or pathological conditions. For example, opening of the BBB's tight junctions may occur under normal conditions to allow the passage of growth factors and antibodies into the brain and in inflammation and can contribute to brain oedema.

**Figure 1:**
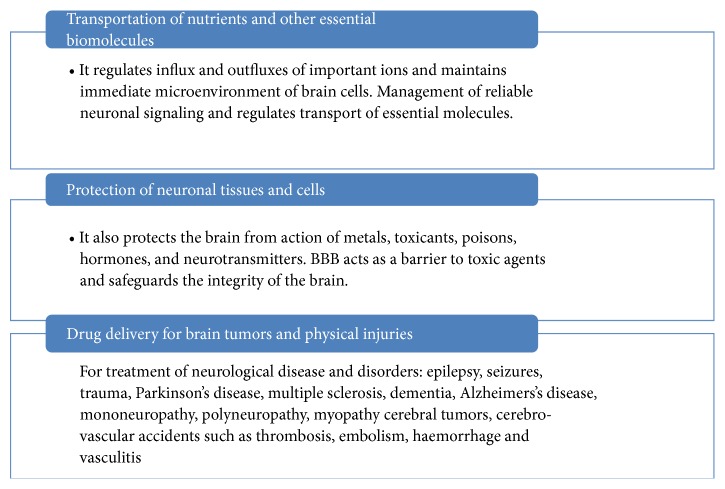
Showing important applications of transendothelial transport through BBB.

The blood brain barrier provides a layer of protection for the brain from harmful or foreign substances that may injure the brain [11]. It also protects the brain from action of metals, toxicants, poisons, hormones, neurotransmitters, drugs, and other foreign or xenobiotic substances [12] mainly noxious agents [13]. It regulates the movement of ions and molecules [14] between the blood and CNS (Figure 1). The barrier is also crucial and provides appropriate environment for proper neural functions as well as for protection of CNS from injury and diseases. Therefore, before targeting the neurovascular unit, protection of the BBB is essential instead of a classical neuron-centric approach that only roads development of neuroprotective drugs that may result in improved clinical outcomes after neural diseases [15] (Figure 1). Hence, for healthy functioning of the brain easy passage of molecules and ions pass through brain barrier (BBB) is highly needful. More often, all essential vital metabolites are to be passed through the BBB to protect the neurocompartments from potential disruptive and damaging xenobiotic agents [16]. Moreover, role of BBB is more important for maintaining normal physiology of brain and for safety of neuronal tissues from toxic substances by restricting its entry into the neurocompartment (Figure 1) [16].

## 2. Functions

**Figure 2:**
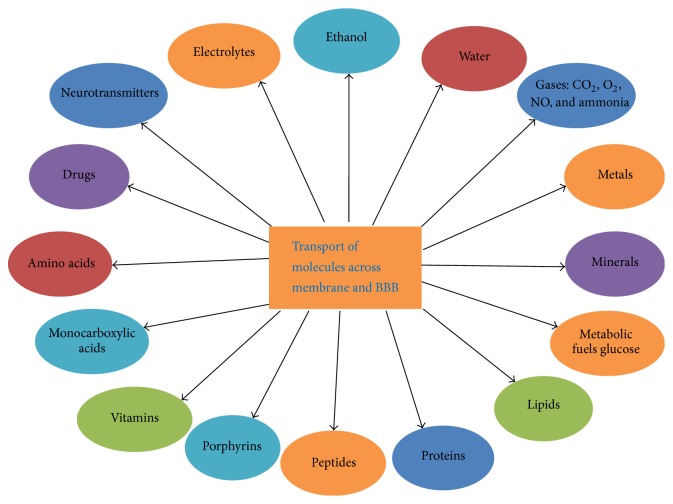
Showing transport of various biomolecules and ions to central nervous system.

BBB acts as a transport barrier that allows passage of selective molecules into the brain and mediating solute flux. It also acts as a dynamic interface which separates the brain from the circulatory system. It limits the entry of biomolecules, serves as a “metabolic barrier”, and allows passage of intracellular and extracellular enzymes and neutral amino acids. It facilitates the entry of metabolically, required nutrients into the brain, and excludes or effluxes potentially harmful compounds or lipid-soluble molecules mainly toxicants [14]. It functions as a perfect barricade to check the permeability of unspecified and unwanted molecules from entering the brain. But, it allows transport of most essential molecules across the membrane and maintains the homeostasis in the most vital organs of the human body. Normally, it allows transport of water, carbon dioxide, oxygen, and most lipid soluble substances such as alcohol, aesthetics but it is impermeable to plasma proteins and non-lipid soluble substances in both cerebrospinal fluid and parenchyma of brain (Figure 2). The barrier shows slight permeability to the electrolytes such as sodium chloride and potassium. Capillary wall possesses three classes of specialized “efflux pumps” which bind to three broad classes of molecules, that is, water-soluble, lipid soluble, and gaseous compounds. Inversely, BBB transports back essential molecules into the blood and transports them to the brain. Therefore, it is highly important and essential that water-soluble compounds including the fuel molecules such as glucose for energy production and amino acids for protein synthesis must cross the BBB (Figure 1).

**Figure 3:**
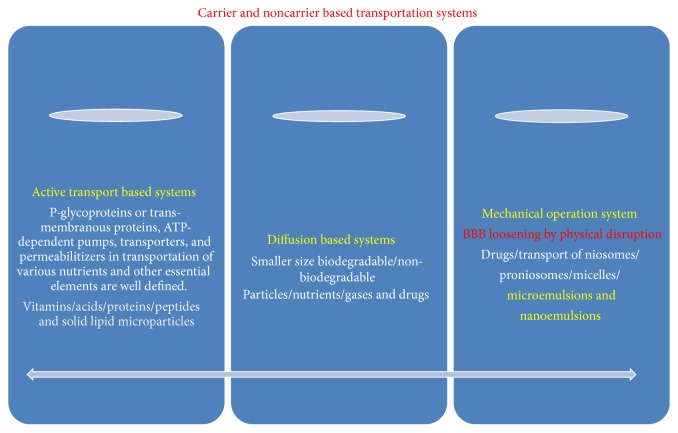
Showing various transport systems used for transportation of molecules and ions.

**Figure 4:**
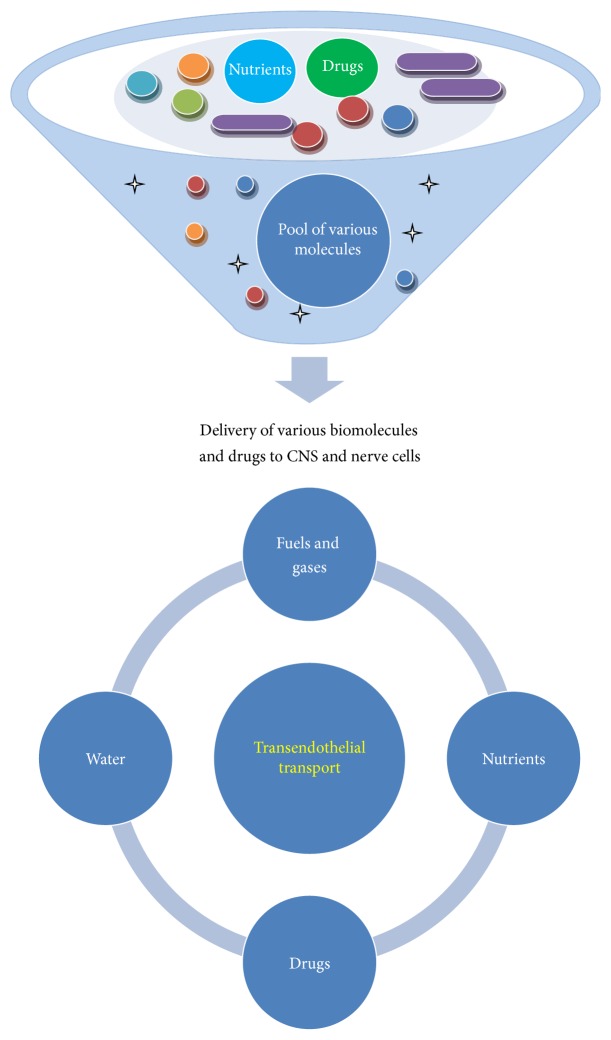
Showing diagrammatic representation of BBB and its therapeutic applications.

The blood brain barrier (BBB) and blood cerebrospinal fluid barrier (BCSF) possess a variety of carrier-mediated transfer systems to support and protect brain function [9]. But, for major function of homeostasis, brain vessels have special carriers on both sides of the cells forming the capillary walls, which transport these substances from blood to brain. Same carriers also remove waste products and other unwanted molecules in the opposite direction. In addition, low pore size disallows entry of biomolecules into the brain (Figure 3). More often, endothelial capillaries in the brain lack endothelial transport, and disallow large molecules to pass but smaller molecules can pass through it. Only glucose and other low molecular fat-soluble molecules are also disallowed but few lipid soluble molecules such as barbiturate drugs rapidly cross through into the brain. More specifically, endothelial light junctions obstruct the movement of polar molecules, but most of the lipophilic substances such as oxygen, carbon dioxide diffuse across the brain or molecules having smaller size or by passive diffusion. Thus, endothelial cells directly or indirectly protect the brain by disallowing the harmful toxic substances and catabolize harmful drugs by using drug metabolizing enzyme system. BBB accomplishes vital functions by maintaining in- and outflow of certain biomolecules (Figure 4). The BBB is semipermeable and allows only few molecules to cross but prevents xenobiotics from entry into the brain and protects it from noxious agents [13]. Thus, BBB plays important role in management of endogenous and xenobiotic compounds by the brain and provides a layer of protection for the brain from harmful or foreign substances that may injure the brain. It also protects the brain from action of metals, toxicants, poisons, hormones, and neurotransmitters (Figure 1). Meanwhile, excess or entry of harmful compound is denied by special mechanisms and structures; possibly, these can diffuse straight through capillary walls into the brain. These are stopped to move through the tight junctions, found between membranes of adjacent endothelial cells.

More specifically, three different barrier layers limit and regulate molecular exchange at the interfaces between the blood and the neural tissue or its fluid spaces: the BBB between the blood and brain interstitial fluid, the choroid plexus epithelium between the blood and ventricular cerebrospinal fluid (CSF), and the arachnoid epithelium between the blood and subarachnoid CSF. These are used for substances with a high lipid solubility and may move across the blood brain barrier by simple diffusion. More specifically, movement of molecules with high electrical charge is slowed down. Morphologically, the polar distribution of transport proteins mediates amino acid homeostasis in the brain. Furthermore, endothelial cells that line cerebral microvessels also maintain microenvironment for reliable neuronal signaling and regulate transport of essential molecules. BBB separates the pools of neurotransmitters and neuroactive agents which act centrally and peripherally. It also regulates influx and outfluxes of important ions and maintains immediate microenvironment of brain cells (Figure 1).

In brain capillary endothelium contains tight junctions which are tighter and more complex in comparison to endothelium found in other tissues. It is polarized into luminal and brain facing plasma membrane and transduces signals from the vascular system and from the brain. This allows molecules to pass through the cell membranes of endothelial cells and reach the brain [17]. More specifically, lipid soluble molecules or micelles could pass through this varying structure, while lipid insoluble molecules are restricted from transport. More exceptionally, few molecules like glucose, oxygen, and carbon dioxide also actively transported across the barrier. These molecules perform various cellular functions mainly cell signaling and communication in the brain. In this mechanism important contribution is made by the transmembrane proteins occludin and the claudins.

BBB also limits the transfer of virus from blood to brain and protects the invasion of virus. Thus, it acts as an anatomical barrier and avoids invasion of various pathogenic infections, toxic effects of drugs, and harmful wastes. Indirectly, BBB assists in maintaining immune responses and protective functions. BBB greatly limits the efficacy of many neuroprotective drugs [18], but it allows the permeability of arsenicals, molybdate and methyl mercury [19], and toxic metals. BBB also limits the usefulness of the most effective chemotherapeutic agents [20]. It is only possible due to presence of an elaborate and dense network of capillary vessels that feeds the brain and removes waste products [21, 22]. Nevertheless, several disorders and diseases can affect the brain leading to some loss of BBB integrity [23]. Therefore, in a state of neuropathological diseases or virus pathogenesis, neural protection can be made by altered BBB functions for drug delivery. Moreover, slight changes by loosening the tight junction could provide good delivery of drugs. Hence, in present review article, endothelial transport and its important role in drug delivery for therapeutics and clinical care of CNS related diseases have been widely elucidated in detail.

## 3. Endothelial Transport

Due to selective and controlled transport of molecules, there occurs a concentration difference in several important constituents in cerebrospinal fluid from ECF (extracellular fluid) that occurs in the body parts. This is due to few cellular associations glued together to form physical obstructions which separate the blood components from ECF and ISF. Normally, concentration difference on both sides is physically controlled by certain physical barriers which prohibit large molecules from coming across the endothelial capillaries or from blood into the cerebrospinal fluid or into the interstitial fluid occuring in the brain. These barriers are blood cerebrospinal fluid barrier and the blood brain barrier that exist between blood and the cerebrospinal fluid and brain fluid, respectively. But, all these components occur in the interstitial fluids of the body. Meanwhile, in the capillary beds of most of the organs, rapid passage of molecules takes place from the blood through the endothelial wall of the capillaries into the interstitial fluid. It plays important role in maintaining composition of interstitial fluid much like that of blood. Further, permeability of important molecules also keeps plasma membrane receptors and transporters of the cells bathed into the interstitial fluid and allows direct interactions with amino acids, vitamins, hormones, proteins, or other compounds of the blood. Under normal conditions, the BBB acts as a barrier to toxic agents and safeguards the integrity of the brain. It is highly selective for transport of nutrients, gases, few peptide, neurotransmitters, and other ions of physiological use and is less toxic or nonharmful to the brain. Most molecules cannot diffuse across the BBB or through a pure phospholipid bilayer at rates sufficient to meet cellular needs, except two gases like CO_2_ and O_2_, and small hydrophobic molecules.

BBB functions as a semipermeable membrane and disallows passage of large molecular substances from the blood into the cerebrospinal fluid or into the interstitial fluids of the brain, even though these same substances are readily transfered into the interstitial fluids of the body. Hence, barriers exist both at the choroid plexus and at the tissue capillary membranes essentially in all areas of the brain parenchyma except in some areas of the hypothalamus, pineal gland, and area postrema where substances diffuse with ease into the tissue space. More specifically, simple diffusion occurs in these areas that are quite important because most of sensory receptors are attached to these regions which quickly respond to specific changes in the body fluids; mainly, changes occur in osmolality and glucose concentration. Further, these responses provide the signals for nervous and hormonal feedback regulation of each of the factors. In general, the blood cerebrospinal fluid and blood brain barriers are highly permeable to water carbon dioxide oxygen and most lipid soluble substances such as alcohol and aesthetics. These are slightly permeable to the electrolytes such as sodium chloride and potassium and impermeable to plasma proteins and most nonlipid soluble large organic molecules. Therefore, the blood cerebrospinal fluid and blood brain barriers often make it impossible to achieve effective concentrations of therapeutic drugs such as protein antibodies and nonlipid soluble drugs in the cerebrospinal fluid or parenchyma of brain. These also remove out harmful blood toxicants and drug metabolites out of the brain. More specifically, all essential molecules are transported across the barrier, while harmful molecules are denied entry, and BBB performs a very effective job of maintaining homeostasis for the most vital organ of the human body [24].

In addition, there are few drugs/compounds which increase the permeability of the blood brain barrier [25] temporarily by increasing the osmotic pressure in the blood which loosens the tight junctions between the endothelial cells. By loosening the tight junctions, restrictiveness of the barrier could decrease and makes it easier to allow a molecule to pass through it. But, this should be done in a very controlled environment because of the risk associated with these drugs. First, the brain can be flooded with molecules that are floating through the blood stream usually blocked by the barrier. Secondly, when the tight junctions loosen, the homeostasis of the brain can also be thrown off, which can result in seizures and the compromised function of the brain [25]. This method is applicable for diffusion for psychoactive drugs. But, rate of entry of compounds that diffuse into the brain depends on their lipid solubility. Moreover, substances with high lipid solubility may move across the blood brain barrier by simple diffusion, while lipid insoluble compounds are denied. For example, the permeability of lipid-soluble compounds, such as ethanol, nicotine, iodoantipyrine, and diazepam, is very high; hence, their uptake by the brain is limited only by blood flow. In contrast, polar molecules, such as glycine and catecholamines, enter the brain very slowly, thereby isolating the brain from neurotransmitters in the plasma.

### 3.1. Transport of Molecules and Ions Across Biomembrane

More specifically, transport proteins are transmembrane proteins which contain multiple membrane spanning segments and are generally α helix. These proteins lined the membrane and allow movement of hydrophilic substances without coming into contact with the hydrophobic interior of the membrane. Oppositely, small hydrophobic molecules pass through the membrane by simple diffusion. Meanwhile, few gases like O_2_ and CO_2_ and small unchanged polar molecules such as urea and ethanol can move by simple diffusion across the membrane (Figure 3). No metabolic energy is required for diffusion because these molecules simply move from a high to a low concentration in a gradient manner. Thus, plasma membrane regulates the transport of molecules into and out of the cell by simple diffusion and active transport (Figure 4).

Brain capillary endothelial cells contain three classes of transmembrane proteins which mediate transport of molecules, ions, sugars, amino acids, and other metabolites across cell membranes. Most of these transport proteins are integral membrane proteins, which remain embedded in the plasma membrane and other membranes. These contain multiple transmembrane domains which permit the controlled and selective transport of molecules and ions across the membrane. These transmembrane proteins couple the energy released by hydrolysis of ATP with the energy required for transport of substances against their concentration gradient. Different classes of pumps belong to transmembrane protein family, exhibit characteristic structural and functional properties, and act as ATP powered pumps or cassettes, channels, and transporters. These also work as ATP-dependent efflux pump and are member of intrinsic membrane proteins like P-glycoprotein (P-gp) [26, 27]. This pump also occurs in the luminal plasma membrane of BMEC and prevents the intracellular accumulation of an extensive variety of chemotherapeutic agents and hydrophobic compounds.

Cytoplasmic matrix contains various kinds of ions which assist in maintaining osmotic pressure and acid base balance in the cells. Retentions of ions in the matrix produce an increase in osmotic pressure that allows entrance of water in the cell. The concentration of ions in the intracellular fluid differs from interstitial fluid. Hence, a high order of difference exists between intracellular K^+^ and extracellular Na^+^ in nerve and muscle cells. Therefore, free calcium ions may occur in cells or circulating blood, while free ions of phosphate (HPO_4_^−^ and H_2_PO_4_^−^) occur in the matrix and blood. These ions maintain buffering system and tend to stabilize pH of blood and cellular fluids. Thus, electrolytes play a vital role in the maintenance of osmotic pressure and acid base equilibrium in the matrix. Mg^2+^ ions, phosphate, and so forth are good examples of the electrolytes. Electrolytes like Na^+^ and Cl^−^ move across by specific channels and transport proteins (Table 1). More exceptionally, few important minerals occuring in matrix in nonionizing state are Na, K, Ca, Cu, I, Fe, Mn, Mo, Cl, Zn, Co, Ni, and so forth because of their much essential physiological need. Thus, the composition of interstitial fluid resembles that of blood, and specific receptors or transporters in the plasma membrane of the cells being bathed by the interstitial fluid may interact directly with amino acids, hormones, or other compounds from the blood (Table 1). More often, barrier limits the accessibility of blood-borne toxins and other potentially harmful compounds to the neurons of the CNS because of restriction imposed by transcapillary movement of substrates in the peripheral circulation into the brain.

### 3.2. Transport of Fuels

Glucose is main primary energy substrate or metabolic fuel of the brain. This maintains major metabolic functions and physiology of neuronal network present inside brain and its regular supply is essentially required. Glucose is a polar substrate whose combustion or catabolism needs large oxygen consumption. Interestingly, glucose transport occurs through both endothelial cells by facilitated diffusion and by GLUT 1 and GLUT 3 transporters [44, 45] present on the surface of neurons and endothelial cells. Normally, brain capillary endothelial cells possess insulin-independent, GLUT-1 glucose transporters which mediate the facilitated diffusion of glucose through the blood brain barrier [44, 46]. Moreover, rate of glucose transport through endothelium depends on energy metabolism occurring inside brain or depends on glucose utilization. Thus, glucose itself functions as a rate limiting ligand or metabolite and it is transferred from ECF to neurons when its concentration falls in blood lower than the normal glucose level. When cellular concentration of glucose becomes high, its transport is obstructed automatically. Further, transporters found on the surface of neuronal and endothelial cells differ in their activity. In fact, same transporters also occur on other cell membranes which transport two to three times more glucose than normally it is metabolized by the brain. Thus glucose utilization depends on concentration resides with the cell that also determines its transport. There are several glucose transporter syndromes known. But among all most common syndromes glucose transporter type 1 deficiency syndrome is important because it more severely effect glucose transport in children. Thus, impairment of GLUT-1 results in a low glucose concentration in the CSF and causes hypoglycorrhachia with clinical symptoms like seizures, mental retardation, and compromised brain development in children. Moreover, most mammalian cells express GLUT1 uniporter transporter that transports blood glucose to fulfill cellular energy needs. This uniporter (GLUT1) transporter traverses between two conformational sites or posses two phases one a glucose binding site faces the outside of membrane while glucose binding site faces inside (Table 2), during the unidirectional transport of glucose from the cell exterior inward to the cytosol. Hence, in a state when glucose concentration becomes higher inside the cellular outside, GLUT1 catalyze the net export of glucose from the cytosol to the extracellular medium. More specifically, it is the stereo specificity of the glucose transport system that permits transport of d-glucose, rather than l-glucose, to enter the brain. Similarly, hexoses, such as mannose and maltose, are rapidly transported into the brain, while the galactose uptake takes place intermediately, and fructose uptake occurs very slowly. Interestingly, uptake of 2-deoxyglucose occurs so fast that it competitively inhibits the transport of glucose. Further, soon after glucose uptake and its transport into the cells, it is rapidly phosphorylated to form glucose 6 phosphate which cannot be transported out of the cell. This step completes more rapidly and at a constant rate. Further, even if intracellular glucose level is low, then glucose is imported from the medium. Sometime, the rate of glucose transport into the ECF normally exceeds the rate required for energy metabolism by the brain. Thus, glucose transport becomes rate limiting if blood glucose levels fall lower than the normal range. In such hypoglycemic condition, glucose level lowers approximately to 60 mg/d which also reduced the *K*_m_ values of the GLUT-1 transporters found in the endothelial cells (Table 2). Moreover, monosaccharide transport proteins play important role in carbohydrate assimilation, distribution, metabolism, and homeostasis [46] (Figures 3 and 4).

### 3.3. Transport of Monocarboxylic Acids

Monocarboxylate transporters play important role in the maintenance of the glycolytic metabolism through proton linked transport of monocarboxylic acid [60] (Figure 2). These are transported by a separate stereospecific system [61] that is slower than the transport system for glucose. In the cerebrovascular endothelium, monocarboxylic acid transporter 1 (Mct1) controls blood-brain transport of short chain monocarboxylic acids such as L-lactate, acetate, pyruvate, ketone bodies, acetoacetate and *β*-hydroxybutyrate monocarboxylic and ketoacids to support energy metabolism and play potential role in treating brain diseases (Table 2) [66]. Monocarboxylate transporter (MCT) isoforms 1–4 catalyze the proton-linked transport of monocarboxylates such as L-lactate across the plasma membrane, whereas MCT8 and MCT10 transporters transport thyroid hormone and aromatic amino acids [61]. Furthermore, MCTs 1–4 also play important metabolic roles including energy metabolism in the brain, skeletal muscle, heart, tumor cells, T-lymphocyte activation, gluconeogenesis in the liver and kidney, spermatogenesis, bowel metabolism of short-chain fatty acids, and drug transport. These transporters showed their distinct properties, expression profile, and subcellular localization matching the particular metabolic needs of a tissue (Table 2). Mct1 function is acutely decreased in rat brain cerebrovascular endothelial cells by *β*-adrenergic signaling through cyclic adenosine monophosphate (cAMP). It constitutively cycles through clathrin vesicles and recycling endosomes in a pathway that is not dependent on cAMP signaling in endothelial cells. Thus, regulated and unregulated vesicular trafficking of Mct1 in cerebrovascular endothelial cells are highly significant for understanding normal brain energy metabolism, etiology, and potential of therapeutic approaches to treating brain strokes/diseases [66]. Normally, during fasting or starvation, level of ketone bodies in the blood gets elevated, and MCT1 transporter gets upregulated. Moreover in adults and neonates during prolonged starvation ketone bodies serve as important fuels for the brain. This results in an elevation rate and capacity of monocarboxylic acid transport substantially in suckling neonates. Therefore, due to increased metabolic requirements of the developing brain in adults, higher concentrations of monocarboxylic acids are supplied in breast milk that fulfill energy needs of neonates. In addition, metabolism of short-chain fatty acids (SCFA), mainly acetate, appears to occur mainly in astrocytes and its transport in the brain is performed by monocarboxylic acid transporter family (Table 2) [62]. It is inhibited by phloretin, a high-affinity inhibitor that binds to MCT1 and slows down physiological activities [62]. More often, potent and specific MCT1 inhibitors can prevent proliferation of T-lymphocytes that may help to achieve promising pharmacological targets including cancer chemotherapy [46].

### 3.4. Transport of Substances and Gases

Brain and other tissue organs possess various structural barriers that are made up of cellular vasculature monolayer of endothelial cells. These cells form elaborate tight junction complexes that efficiently limit the paracellular transport of solutes. The BBB has a number of highly selective mechanisms for transport of nutrients into the brain. But, there occur four basic mechanisms by which solute molecules move across membranes. First mechanism is simple diffusion, which proceeds from low to high concentrations. Second is facilitated diffusion, a form of carrier-mediated endocytosis, in which solute molecules bind to specific membrane protein carriers. This also controls from low to high concentration. Third is simple diffusion which occurs through an aqueous channel, formed within the membrane. Fourth is active transport through a protein carrier with a specific binding site that undergoes a change in affinity. Active transport requires ATP hydrolysis and conducts movement against the concentration gradient. Moreover, movement between cells is paracellular diffusion [58], while diffusion across the cells is called transcellular diffusion. Both types of diffusion mechanism are found in the brain, both of which are nonsaturable and noncompetitive. It is a spontaneous process depending on random movement of solutes. It occurs due to free-energy change of a solute diffusing across a membrane that directly depends on the magnitude of the concentration gradient. Moreover, para cellular diffusion does not occur to any great extent at the BBB, due to presence of tight junctions or structural barriers. Moreover, para cellular diffusion does not occur to any great extent at the BBB, due to presence of tight junctions or structural barriers but in transcellular diffusion, the general rule is followed that is higher the lipophilicity of a substance; greater will be its diffusion into the brain [59]. More specifically, biogenic amines are transported through BBB by diffusion and it never occurs by carrier-mediated transport. Contrary to this, for uptake of other substances, uptake1 carrier involves that occuring at the luminal surface of BBB. More specifically, if two substances are similar, but vary in molecular weight, the smaller substance will penetrate more rapidly; consequently, small inorganic molecules (i.e., O_2_, CO_2_, NO, and H_2_O) are highly permeable. Additionally, hydrogen bond reduction of a compound will enhance its membrane permeability. Removal or masking of hydrogen bonding donor group from a compound will effectively decrease the transfer energy from water into the cell membrane [47].

More specifically, capillary endothelial cells also possess wide range of transporters that selectively transport solutes and xenobiotics [65]. These transporters are found at the luminal (blood) side of membranes* in vivo* and maintain specific transport mechanisms for transport of various biomaterials [66]. This is the main reason that, the capillary beds most of organs, allow rapid passage of molecules from blood through the endothelial wall of the capillaries into the interstitial fluid. Therefore, diffusion or transport of biomolecules across the membrane is operated with the help of integral and transmembrane proteins and receptors. Meanwhile, transported molecules and ions interact to specific receptors or transporters in the plasma membrane of the cells. These receptors remain embedded or bathed by the interstitial fluid and may interact directly with amino acids, hormones, or other compounds from the transported or transferred from blood. Due to semipermeable nature of BBB, many nonpolar substances, such as drugs and inert gases, directly diffuse through the endothelial cell membranes, while a large number of other compounds are transported through the endothelial capillaries by facilitative transport. Contrary to this, nonessential fatty acids cannot come across the blood brain barrier but essential fatty acids are transported across the barrier. Further, metabolic fuels, monocarboxylic acids, ethanol, vitamins and neurotransmitters are carried by carrier-mediated transport because low lipid soluble biomolecules can traverse the blood brain barrier. But, water and gases like O_2_, CO_2_, and NO_2_ diffuse directly across the endothelial wall (Table 2). Diffusion plays a crucial role in brain function. The spaces between cells can be likened to the water phase of foam and many substances move within this complicated region. Diffusion in this interstitial space quantified from measurements based on novel microtechniques. Besides delivering glucose and oxygen from the vascular system to brain cells, diffusion also moves informational substances between cells, a process known as volume transmission. Therefore, diffusion is also found essential to many therapies that deliver drugs to the brain. The diffusion-generated concentration distributions of well-chosen molecules also reveal or image the structure of brain tissue. These concepts and methods are highly applicable for making therapeutic measures for improvement of neurodegenerative diseases [67].

In state of any infection, concentration of both gases differs largely. Therefore, patients under different physiological and pathological conditions show nonrespiratory acidosis and a decrease in pa_O2_ induced an increase of CBF when the oxygen tension in cerebral venous blood fell below 35–40 mm Hg. At the same time, the cerebral glucose uptake, the cerebral lactate output, and the lactate-pyruvate ratio in cerebral venous blood increased. Critical conditions occurred for the oxygen supply of the brain when cerebral venous p_O2_ fell below approximately 30 mm Hg. In edematous brain areas of patients, rCBF decreased with increasing brain water content. Disease state and invasion of infection largely affect hematocrit, vascular diameter, blood viscosity, blood flow, metabolic rate, nonlinear oxygen dissociation curve, arterial PO_2_, P_50_ (oxygen tension at 50% hemoglobin saturation with O_2_), and carbon monoxide concentration. Finally, the various types of hypoxia such as hypoxic, anemic, and carbon monoxide hypoxia arise [70]. It also affects oxygen transport in brain microcirculation [70]. More often, systemic and myocardial oxygen transport shows responses to brain death in pigs [48]. Both SVR and EO(2) decreased after brain death (*P* < .01) and remained low, while lactate level remains unchanged. Moreover, profound metabolic alterations occur in condition of brain death and major complications are related to oxygen transport and its supply [48].

It is clear that capillary blood flow played an important role in the transport of gases and extra vascular vasomotor action. The exchange of gases such as CO_2_, O_2_, N_2_O, and Xe and volatile anesthetics in cerebral tissues takes place rapidly by simple diffusion (Table 2). This depends on cerebral blood flow and cerebral metabolic rate of oxygen consumption in the cortical capillary levels [71]. Direct external application of oxygen to cerebral cortex causes cerebral vasoconstriction, while carbon dioxide resulted in dilation of cerebral vessels [72]. Further, concentration of these gases in the brain comes into equilibrium with the plasma which is primarily limited by the cerebral blood flow rate. In addition, gaseous exchange brain also depends on pure ventilation and neurological, physiological, and pathological state [73]. Moreover, end tidal carbon dioxide acts as a marker of stress response in patients [73], while inert gases, like N_2_O and Xe, can be used as markers to measure cerebral blood flow. An interesting contrast is observed between CO_2_ and H^+^ with regard to their effects on brain pH. As the permeability of CO_2_ greatly exceeds in BBB than the H^+^, the pH of the brain interstitial fluid reflects blood pCO_2_ rather than blood pH. Interestingly, central respiratory chemoreceptors sense the changes that occur after alteration in CO_2_ concentration and pH in the brain tissue [74]. Consequently, patients which facing metabolic acidosis show compensatory respiratory alkalosis, and their brain contain high pH and become alkalotic. Besides these gases, ammonia is believed to play key role in the development of hepatic encephalopathy with increased formation of glutamine playing a central role. Ammonia passes through BBB by passive diffusion [49], while nitric oxide donor induces long-term survival in rats [50].

### 3.5. Diffusion of Water

Water rapidly enters the brain passing through capillary membranes via the intercellular gaps between or pores in the endothelial cells or special areas where the cytoplasm is very thin. Normally, due to high permeability, water moves freely into or out of the brain as the osmolality of the plasma changes. In addition,cell membrane possesses specific channels or pores through which diffusion of water occurs in association of certain proteins. These proteins are named aquaporins that belong to family of membranous proteins. These proteins allow water and few other small uncharged molecules such as glycerol to cross biomembrane. These proteins are found in all living organisms and at least 6 different water channel proteins have been identified in various cell membranes in humans. These aquaporin proteins form complexes that span the membrane and water moves through these channels passively in response to osmotic gradients (Table 2). These channel proteins are present in highest concentrations in tissues where rapid transmembrane water movement occurs, for example, in renal tubules. Moreover, aquaporin 0 found in the lens in the eye has a role in maintaining lens clarity. While aquaporin 1 occurs on the red cell membrane, and proximal convoluted tubule, thin descending limb of the Loop of Henle in the kidney, choroid plexus, smooth muscle, unfenestrated capillary endothelium, exocrine sweat glands, hepatic bile ducts and gallbladder epithelium where it perform water diffusion. Moreover, aquaporin 2 is ADH-responsive water channel specifically found in the collecting duct in the inner medulla. Furthermore, insertion of the channel into the apical membrane also occurs following ADH stimulation. Aquaporins 3 and 4 normally are found in the basolateral membrane in the collecting duct but they are not altered by ADH levels. Recently, aquaporin 4 has also been identified in the ADH-secreting neurons of the supraoptic and paraventricular nuclei in the hypothalamus that functions as a hypothalamic osmoreceptor and regulates body water balance. Aquaporin 5 occurs in lacrimal and salivary glands and in the lungs. Thus, aquaporins increase the water permeability of cell membranes, while other membranes of aquaporins family transport hydroxyl-containing molecules such as glycerol rather than water. Few structural mechanisms involved in gating induced regulation of aquaporins [75]. Aquaporins or water channel proteins are also found in insects the western tarnished plant bug,* Lygus hesperus* (Table 2) [49] and plants. These proteins control water relations [54] and act as regulators of transcellular water flow [52].

Water also easily enters the lymphatic capillaries via gaps between the lymphatic endothelial cells. These gaps function as flap valves and promote forward lymph flow when the capillaries are compressed. In other areas of the body, the water permeability of capillary membranes is quite low, because pores or slits are lacking in the blood brain barrier and it greatly limits water movement by the intercellular pathway. Hence, water transport occurs by dissolving in the membrane phase. This refers to water crossing the lipid bilayer of the cell membrane by diffusion. But, lipid composition of different cell membranes varies, which determines rate of fluid flow across cell membranes that greatly varies. In some membranes, the water flux is very high and cannot be accounted for water diffusion across lipid barriers. Therefore, it is possible and operative that membranes possess some proteins that form aqueous channel through which water can pass. This is inferred in artificial lipid bilayers; water does not cross membrane easily, and the same process occurs in natural membranes. In addition, substances, which are water-soluble typically, do not cross lipid membranes easily unless specific transport mechanisms are present. Nevertheless, paradoxically, water crosses nearly all the membranes in the body with ease. Moreover, water permeability and transport are regulated by the capillary endothelium and rate of cerebral blood flow. In fact, in normal condition permeability, constant of the cerebral capillary wall to the diffusion of water is about the same as that required for its diffusion across lipid membranes. Thus, osmotic pressure allows water to move across membranes and its unavailability produces deficiencies known as fenestrations and diffusion across the lipid cell membranes of the endothelial cells. Fenestrations are found only in capillaries in special areas where very high water permeability is necessary for the function of these areas. These fenestrated capillaries and vascular sinusoids permit rapid exchange of water and solute between the plasma and extracellular fluid and supply the choroid plexus (Table 2) [53]. High water permeability is maintained in glomerular capillaries found in kidney than in muscle capillaries. Other areas with fenestrations are the capillaries in the intestinal villi and in ductless glands.

### 3.6. Transport of Ethanol

Alcohol consumption causes intoxication of cells, as it is transported into the brain. Once alcohol is consumed, it leaves the gastrointestinal (GI) tract and enters into the bloodstream through blood capillaries. It is carried to the heart by streaming blood, from where alcohol is sent to the lungs and also come back to the heart with the oxygenated blood. Alcohol is pumped through the arterial system and distributed in all organs of the body. Ethanol travels within the arteries that lie between the skull and the brain itself. These arteries branch out into capillaries and dive deep into the brain tissue. Ethanol passes through these capillaries so as to reach the neurons in the brain. Unfortunately, in the brain, there is no barrier for ethanol and it crosses the blood brain barrier very easily. It is a polar solvent, lipophilic in nature that mixes easily with the fat in the membrane. It makes it easy for ethanol to cross the blood brain barrier. In the case of other biological membranes, ethanol moves across by filtration moving through water spaces because it dissolves in water, where it is absorbed by passive diffusion and moves with the concentration gradient through the membrane. Similarly, in the brain capillaries, due to lipophilic property, ethanol allows moving by passive diffusion across the endothelial cell membrane and through the astrocyte layers. Similarly, small lipophilic drugs diffuse passively across the blood brain barrier including nicotine, marijuana, and heroin that cause intoxication in the brain (Table 2). Contrary to this, water-soluble nutrients such as glucose and large water-soluble molecules such as vitamins need specific transporters to be transported across the BBB. This process requires energy and is known as active transport of biomolecules. Increased uptake of alcohol increases neurobiological effects [55] and alters basal extracellular glutamate concentrations and clearance in the mesolimbic system of alcohol-preferring [56]. Alcohol-induced oxidative/nitrosative stress alters brain mitochondrial membrane properties in experimental animals (Table 2) [57].

### 3.7. Transport of Vitamins

Vitamins are indispensable dietary compounds which require in small amounts few micrograms to milligrams per day to be present in our food. These do not serve as an energy supply. They are low molecular weight compounds and their insufficient intake causes malnutrition and multiple metabolic defects and diseases. Vitamin deficiency causes certain physiological and biochemical abnormalities which affect body metabolism, growth, and development. Vitamins cannot be synthesized inside body and are required in small amounts to support normal metabolism. They are needed for normal function, growth, and maintenance of body tissues and for regulation of chemical reactions in the body. Vitamin D affects brain development and function and its low levels cause neuropsychiatric diseases like autistic spectrum disorder and schizophrenia [76]. Vitamin D deficiency in early life affects neuronal differentiation, axonal connectivity, dopamine ontogeny, and brain structure and function (Table 3) [76]. Deficiency of vitamin E may cause neurological dysfunction, myopathies, and diminished erythrocyte life span. Chemically, vitamins belong to diverse classes of compounds and are classified according to their solubility into water and fat. Since vitamins cannot be synthesized by the brain, they must be obtained from the blood. Thus, specific transport systems are required for carrying vitamins across blood brain barrier (Table 3). Generally, these transport systems possess a low capacity since the brain requires only small amounts of the vitamins and efficient homeostatic mechanisms preserve brain vitamin content without the need for a rapid influx from the blood. How vitamins are transferred across the mammalian blood brain barrier and choroid plexus into brain and CSF and how vitamin homeostasis in brain is achieved is an important issue (Table 3) [77].

The majority of vitamins are water-soluble but a few vital vitamins such as A, D, E, and K are fat-soluble. They are absorbed in the lymph and are transported in the blood with carrier proteins, from which these are delivered to the brain. Both fat-soluble and water-soluble vitamins have different mechanism of absorption and transport. The water-soluble vitamins are B and C which are absorbed easily through diffusion (Table 3). The B vitamins include thiamin, riboflavin, niacin, folate, pyridoxine, and B12. The water-soluble vitamins are easily dissolved and can be excreted in the urine. Moreover, vitamin deficiency would only be corrected by ingesting that vitamin through diet. Nevertheless, dietary deficiency of some vitamins can produce neurological disease. Transport and metabolism of vitamin B6 forms are transported across brain and choroid plexus in nonphosphorylated B form [84] and have separate carriers (Table 3) [77]. Moreover, after transport, vitamins are accumulated by brain cells by separate, specialized systems. More often, both cofactor and noncofactor vitamins (e.g., of B(1), B(3), B(6), and E) have potential role in the therapy of brain disorders [77]. In bacteria,* E. coli* vitamin B12 permease is ABC protein transporter that imparts a variety of molecules form the environment. These transport proteins contain two transmembrane domains (T) and two cytosolic ATP binding domains. In mammals, nearly 50 different ABC transporter proteins are known these are mostly expressed in the liver, intestine, kidney, and brain sites where natural toxic and waste products are removed by the body. The main substrates of these ABC transporter proteins are sugar amino acids, cholesterol, bile acids, phospholipids, peptides, proteins, toxins and foreign substances (Table 3). More specifically, some ABC proteins flip phospholipids and other lipid soluble substrates from one membrane leaflet to the opposite leaflet (Table 3).

Vitamin C crosses the blood brain barrier in the oxidized form through the glucose transporters [82]. In contrast, the oxidized form of vitamin C, dehydroascorbic acid (oxidized ascorbic acid), readily enters the brain and is retained in the brain tissue in the form of ascorbic acid. More specifically, glucose GLUT1 also transports dehydroascorbic acid into the brain and its transport is inhibited by d-glucose, but not by l-glucose (Table 3). The facilitative glucose transporter, GLUT1, is expressed on endothelial cells at the blood brain barrier and is responsible for glucose entry into the brain [82]. It is an important mechanism by which the brain acquires vitamin C. Further, oxidation of ascorbic acid is an important regulatory step in accumulation of the vitamin by the brain [82]. Vitamin C concentrations in the brain exceed those in blood by 10-fold. In both tissues, the vitamin is present primarily in the reduced form, ascorbic acid.

Fat-soluble vitamins such as A, D, E, and K are absorbed in the lymph and are transported in the blood with the help of specific carrier proteins, from which these are delivered to the brain.. Transport of vitamins A (retinol, retinoic acid) and D (1,25-dihydroxyvitamin D3 [1,25-(OH)2D3] and 25-hydroxyvitamin D3 [25-(OH)D3]) derivatives through the rat brain capillary endothelial wall, that is, the blood brain barrier (BBB), occurs in two forms (Table 3): first, after binding to albumin and a plasma transport protein to which these vitamins bind with specific high-affinity. Moreover, vitamin D and its hydroxylated metabolites are transported in the blood, bound to a transport protein DBP [79] that is also very important in the placental transfer of 25-hydroxy-vitamin Dl. Moreover, during absorption from the intestine, vitamin A and *β*-carotene become finely dispersed in the intestinal fluid in mixed lipid micelles formed with the aid of bile salts, lysolecithin, lower glycerides, and cholesterol. These facilitate the transfer of the vitamin and other lipid components across the mucosal cell membrane [78]. Interestingly ester is transported in association with *β*-carotene in the *β*-lipoprotein fraction. More specifically, physiologically active vitamin A alcohol on the other hand is attached to a specific protein carrier with properties very similar to ceruloplasmin in the α_2_-globulin fraction (Table 3) [78].

Vitamin E includes eight naturally occurring fat-soluble nutrients called tocopherols and dietary intake of vitamin E activity is essential in many species (Table 3) [80]. α-Tocopherol is absorbed via the lymphatic pathway and transported in association with chylomicrons. In plasma, alpha-tocopherol is found in all lipoprotein fractions but mostly associated with apo-B-containing lipoproteins in man. In rats, approximately 50% of alpha-tocopherol is bound to high density lipoproteins (HDL). After intestinal absorption and transport with chylomicrons, alpha-tocopherol is mostly transferred to parenchymal cells of the liver where most of the fat-soluble vitamin is stored. Very small amount of vitamin E is stored in the nonparenchymal cells (endothelial, stellate, and Kupffer cells). α-Tocopherol is secreted in association with very low density lipoprotein (VLDL) from the liver [80]. In the rat, about 90% of total body mass of alpha-tocopherol is recovered in the liver, skeletal muscle, and adipose tissue. Most α-tocopherol is located in the mitochondrial fractions and in the endoplasmic reticulum, whereas little is found in cytosol and peroxisomes (Table 3) [80].

Normally, vitamin E derived from food is absorbed in the intestine and then transported into the liver on molecules called chylomicrons. After a meal, chylomicrons are formed to transport fat-soluble vitamins (such as vitamin E), dietary fats, and cholesterol from the intestine to the liver. Once in the liver, αTTP transfers vitamin E from chylomicrons to very low-density lipoproteins (VLDLs), which carry fat, fat-soluble vitamins, and cholesterol from the liver to other tissues throughout the body. The VLDLs are then released into the bloodstream so the accompanying vitamin E can be used in the body. The αTTP protein is also thought to transport vitamin E to nerve cells (neurons) in the brain. Vitamins or their derivatives perform important roles as catalysts of enzymatic reactions; hence, they are also known as coenzymes. Biotin, pyridoxal-phosphate, and riboflavin can be covalently bound to the apoenzymes and are called prosthetic groups. Other coenzymes, in contrast, serve as mobile carriers of reactive chemical compounds. Most reactions of primary metabolism require the help of vitamins. The TTPA gene provides instructions for making the α-tocopherol transfer protein (αTTP), which is found in the liver and brain. This protein controls the distribution of vitamin E obtained from the diet (also called α-tocopherol) to cells and tissues throughout the body. Vitamin E is an antioxidant that protects cells in the body from the damaging effects of unstable molecules called free radicals (Table 3).

Quinone compounds act as membrane resident carriers of electrons between components of the electron transport chain in the periplasmic space of prokaryotes and in the mitochondria of eukaryotes. Vitamin K is a quinone compound in the human body in a storage form as menaquinone (MK); distribution includes regulated amounts in mitochondrial membranes. The human brain, which has low amounts of typical vitamin K dependent function (e.g., gamma carboxylase), has relatively high levels of MK, and different regions of brain have different amounts [81]. Coenzyme Q is a quinone which synthesizes* de novo*, and its levels of synthesis decline with age. The levels of MK are dependent on dietary intake and generally increase with age. MK has a characterized role in the transfer of electrons to fumarate in prokaryotes. A newly recognized fumarate cycle has been identified in brain astrocytes (Table 3) [81].

Slc23a1 is ascorbic-acid transporter that is essential for vitamin C transport into the brain, many tissues (Table 3). Vitamin C crosses the placenta and is essentially required in the perinatal period (Sotiriou et al, 2002) [83]. It is used to prevent scurvy and works as a cofactor for hydroxylases required for posttranslational modifications that stabilize collagen. In addition, vitamin C is also essential for performing many enzymatic reactions. Vitamin C also acts as a free radical scavenger. Moreover, few specific nonoverlapping transport proteins mediate the transport of the oxidized form of vitamin C, dehydroascorbic acid, and the reduced form, L-ascorbic acid, across biological membranes. Vitamin C also crosses the blood-brain barrier in the oxidized form through the glucose transporters [82] (Table 3). Dehydroascorbic acid uptake occurs by facilitated-diffusion glucose transporters, GLUT 1, 3, and 4, but under physiological conditions. These transporters are unlikely to play a major role in the uptake of vitamin C due to the high concentrations of glucose that effectively block its influx. In addition, L-ascorbic acid enters cells via Na^+^-dependent systems that have two isoforms SVCT1 and SVCT2v transporters. Transport by both isoforms is stereospecific, with a pH optimum of approximately 7.5 and a Na^+^ and ascorbic acid stoichiometry of 2 : 1. SVCT2 may exhibit a higher affinity for ascorbic acid than SVCT1 but with a lower maximum velocity [83]. The two isoforms also differ in their tissue distribution: SVCT1 is present in epithelial tissues, whereas SVCT2 is vitamin C transport systems of mammalian cells occuring in most tissues with the exception of lung and skeletal muscle and mammalian cells (Table 3) [85].

### 3.8. Transport of Neurotransmitters

Neurotransmitters are small, water-soluble molecules which are diffused across the membrane of presynaptic and post synaptic neuron fiber. There are three main categories of substances that act as neurotransmitters. First category is amino acids such as glutamic acid, GABA, aspartic acid, and glycine; second category is of peptides such as vasopressin, somatostatin, and neurotensin; third category is of monoamines like norepinephrine, dopamine, serotonin, and acetylcholine. Among all neurotransmitters, glutamic acid (=glutamate) and GABA are major neurotransmitters of the brain but monoamines and acetylcholine perform specialized modulating functions, often confined to specific structures. The peptides neurotransmitters perform specialized functions in the hypothalamus and act as cofactors elsewhere in the brain. Based on chemical nature and receptor binding brain, neurotransmitters are different and perform varied functions. For example, glutamate performs excitatory function, whereas others (like GABA) are primarily inhibitory (Table 4). Dopamine interacts with receptors and shows either excitatory or inhibitory effect. These are receptors which determine whether a transmitter acts rapidly by direct action on an ion channel (e.g., nicotinic acetylcholine receptors) or slowly by a second-messenger system that allows for synaptic plasticity (e.g., muscarinic acetylcholine receptors). Interestingly, both speed and mechanism of transmitter inactivation after the signal also act as a factor. In addition, different neurotransmitters such as acetylcholine, serotonin, and catecholamine are synthesized, released, transported, inactivate, and signal in different manner. These widely differ in delivery in neuronal circuits located in the brain (Table 4) [86].

More often, CNS contains many neurotransmitters but peripheral nervous system contains acetylcholine and norepinephrine neurotransmitters. Acetylcholine is a major neurotransmitter that occurs in the peripheral nervous system. It is usually but not always an excitatory neurotransmitter in contrast to the monoamine neurotransmitters, which are nearly always with a few exceptions inhibitory. Acetylcholine is synthesized in the brain from acetyl-CoA and formed in glucose metabolism and is in association with choline, which is actively transported across the blood brain barrier. The acetyl-CoA and choline are independently synthesized in the neuron cell body (motor neurons). Most dietary choline comes from phosphatidyl choline (Table 4), the major phospholipid in the membranes of plants and animals, but it does not occur in bacteria. Acetylcholine is independently transported along the axon to the synapse where they are conjugated into acetylcholine. Acetylcholine receptors are located on the surface of post synaptic cell membrane of nerve cells of brain as well as outside the brain on muscle cell surface where it controls excitation. Acetylcholine is transported through receptor-mediated transport either by ligand gated channels or by G protein coupled receptor receptors. Similarly, choline is also transported carrier-mediated process. Acetylcholine is largely inhibited by certain molecules such as dimethyl aminoethanol, hemicholinium, and tetraethyl ammonium chloride. As it is true that the brain and its blood brain barrier cannot synthesize choline, its transport occurs by transporters which regulate the formation of acetylcholine in the central nervous system (Table 4) [87]. Moreover, binding of neurotransmitters to a G protein coupled receptor induces the opening and closing of a separate ion channel protein over a period of seconds or minutes. More exceptionally, neurotransmitters, mainly opioids, are directly released from the brain to the blood through saturable glycoproteins (Pgp) transport system [88]. It is also true that all neurotransmitters are released by exocytosis, which involves certain molecular events and protein complexes [86]. Moreover, bisazaaromatic quaternary ammonium salts act as ligands for the blood brain barrier choline transporter [89], by exploiting nutrient transporters at the blood brain barrier, and can improve brain distribution of small molecules [90], while stress induces choline transport across blood barrier (Table 4) [91].

#### 3.8.1. Amino Acid Neurotransmitters

Glycine is a neurotransmitter that is only found in vertebrate animals. After synthesis, it is released into a synapse and binds to a receptor which makes the postsynaptic membrane more permeable to Cl^−^ ions. Thus, glycine hyperpolarizes the membrane and makes it less likely to depolarize. It is an inhibitory neurotransmitter (Table 4). Aspartate is neurotransmitter that is primarily localized to the ventral spinal cord and opens an ion channel. It is inactivated by reabsorption into the presynaptic membrane. Aspartate is an excitatory neurotransmitter, which increases the likelihood of depolarization in the postsynaptic membrane. Moreover, both glutamic acid and aspartic acid are the two acidic amino acids found in proteins which possess 2 carboxyl groups. These amino acids destroy neurons when released in excessive amounts. Interestingly, both of these form an excitatory/inhibitory pair in the ventral spinal cord comparable to the excitatory/inhibitory pair formed by glutamate and GABA in the brain. Glutamate is the most common neurotransmitter in the brain. It is always excitatory, because of simple receptors that increase the flow of positive ions by opening ion channels. Glutamate stimulation is terminated by a (chloride-independent) membrane transport system that is only used for reabsorbing glutamate and aspartate across the presynaptic membrane. Glutamate and aspartate reenter the cell by a transporter driven by the high extracellular concentrations of Na^+^ and the high intracellular concentrations of K^+^. Sodium enters the cell along with the amino acids, while potassium leaves the cell in similar proportion. Thus, entry of both glutamate and asparate is indirectly powered by the ATP-driven Na^+^-K^+^-ase (sodium pump) which creates the high ion concentration gradients (Table 4). As aspartate is an essential amino acid that does not synthesize in biological systems. Hence, it is either supplemented in the diet as synthetic chemical or supplied by any exogenous source.

Aspartate binds specifically to the NMDA glutamate receptor which is regulated both by a glutamate molecule and by voltage. NMDA receptors interact with ligand repeatedly and develop capacity for an activity-dependent increase in synaptic efficiency that work as long-term potentiation or LTP, which plays important role in learning and memory. NMDA receptors are found densely concentrated in the cerebral cortex mainly in hippocampus (CA1 region) amygdala and basal ganglia. These are highly vulnerable to glutamic acid that makes damaging effects due to excessive excitatory neurotransmitter release. Similarly, excitotoxicity due to glutamic acid is a major destructive process seen in stokes and other forms of brain ischemia. Moreover, granule cells of the dentate gyrus of the hippocampus are rich in nitric oxide synthetase. Glutamate stimulation of NMDA receptors results in nitric oxide synthesis and enhancing neurotransmitter release from adjacent synapses. Here, nitric oxide works as neuromodulator (Table 4). Similarly, monosodium glutamate (MSG) occurs as a major component of soya sauce and destroys nerve cells when fed to young animals. Normally it does not cross the blood-brain barrier but functions as a neurotransmitter is an important question. Increased alertness (or anxiety) due to caffeine may be mainly due to blockage of adenosine receptors which normally inhibit glutamate release. In turn, glutamate released into synapses is either reabsorbed directly into neurons by the ion-exchange transport system or is soaked up by astrocytes or glial cells which convert the glutamate into glutamine a molecule which cannot cause excitotoxicity. The glutamine can then be safely transported back to neurons for reconversion into glutamate. One of the damaging effects of mercury poisoning is swelling of astrocytes, which are rendered unable to soak up glutamine from synapses contributing to excitotoxicity (Table 4).

#### 3.8.2. Gamma Amino Butyric Acid (GABA)

GABA is the major inhibitory neurotransmitter of the brain, occurring in 30–40% of all synapses. Its concentration in the brain is 200–1000 times greater than that of the monoamines or acetylcholine. It occurs highly concentrated in the substantia nigra and globus pallidus nuclei of the basal ganglia, followed by the hypothalamus, the periaqueductal grey matter, and the hippocampus. GABA receptor is connected to a chloride ion channel that allows more chloride ions to enter inside the cell and make the membrane less likely to depolarize. GABA is synthesized in the brain from the Krebs citric acid molecule, that is, α keto glutarate (Table 4). GABA is commonly inactivated after release into the synapse by active transport into the astrocyte glial cells that are closely associated with synapses. GABA is synthesized from glutamic acid and is catabolized back into the citric acid cycle. The vitamin B6 derivative pyridoxal phosphate is a cofactor in the synthesis of GABA. It imposes inhibitory effects on the brain neurons that show protective role during hypoxia or ischemia. Both alcohol and barbiturates show similar effects on the GABA receptors. In fact, potentiation of chloride influx into neurons is a major mechanism in the effect of ethanol on the brain (Table 4).

#### 3.8.3. Primary Monoamine Neurotransmitters

Dopamine, norepinephrine, and serotonin are primary monoamine neurotransmitters. Biochemically, both dopamine and norepinephrine are catecholamines, while serotonin is an indolamine. Both dopamine and epinephrine are primarily inhibitory neurotransmitters and make postsynaptic cells inhibitory for catecholamines. There are 3-4 times more dopaminergic cells in the CNS than adrenergic cells. Dopamine in the caudate nucleus facilitates posture, whereas dopamine in the nucleus is found associated with animal's speed and pleasure. There are two primary dopamine receptor types: D_1_ (stimulatory) and D_2_ (inhibitory); both act through G-proteins D_2_ receptors (Table 4). More often, these occur on the dopaminergic neurons and provide partial negative feedback. These are autoreceptors that can inhibit both dopamine synthesis and release. Moreover, amino acid tyrosine is converted into dihydroxyphenylalanine (DOPA) by the tyrosine hydroxylase enzyme using oxygen, iron, and tetrahydrobiopterin (THB) that act as cofactors. High concentrations of dopamine inhibit tyrosine hydroxylase activity through an influence on the THB cofactor. There are four main dopaminergic tracts in the brain the nigrostriatal tract, tuberoinfundibular tract, mesolimbic tract, and mesocortical tract. Dopamine cells project topographically to the areas they innervate. Overstimulation of D_2_ receptors in the mesolimbic and mesocortical systems evokes schizophrenia. Dopamine is also responsible for induction of vomiting by stimulation of D_2_ cells in the chemoreceptor trigger zone, stimulation of growth hormone release by D_2_ receptors, and increased exploration and locomotion. Interestingly, in the males, sexual behavior is increased by dopamine agonists, whereas sexual behavior in females is increased by dopamine antagonists. Moreover, for treatment of Parkinsonian patients DOPA, treatment is given but it develops psychotic symptoms resembling schizophrenia (Table 4).

*Norepinephrine*. Norepinephrine is a major neurotransmitter that occurs in the peripheral nervous system. It is synthesized from dopamine means of the enzyme dopamine beta-hydroxylase (DBH), with oxygen, copper, and vitamin C as cofactors inside adrenal medulla. Dopamine synthesis occurs in the cytoplasm, but norepinephrine is synthesized in the neurotransmitter storage vesicles. Cells which utilize norepinephrine for formation of epinephrine use s-adenyl methionine (SAMe) as a methyl group donor, an elevated level of cortisol in the medulla, induce phenylethanolamine N methyltransferase (PNMT), an enzyme which catalyzes the conversion of norepinephrine to epinephrine. The most prominent norepinephrine-containing (noradrenergic) nucleus identified is locus ceruleus located in the pons, which accounts for over 40% of noradrenergic neurons in the rat brain. Other noradrenergic neurons are clustered in lateral tegmental area, neocortex, hippocampus, and cerebellum that receive noradrenergic stimulation exclusively from the locus ceruleus (Table 4). Most of the dopaminergic innervation of the hypothalamus comes from the lateral tegmental nuclei.

Epinephrine plays important role in short-term and long-term regulator of stress and development of illness [106]. It occurs in much lesser amount than norepinephrine. Desipramine inhibits norepinephrine reuptake, with little effect on dopamine. Similarly, imipramine and amitriptyline are inhibitors of norepinephrine and serotonin reuptake by the presynaptic terminals but are more potent for serotonin. Beta-noradrenergic receptors also apparently inhibit feeding, whereas alpha-receptors seem to stimulate feeding. Moreover, MAO inhibitors reduce metabolism of all catecholamines; it is believed that the antidepressant effect is more related to norepinephrine than to dopamine. More specifically, tricyclic antidepressants such as amitriptyline 3-ring structure increase appetite that is considered as a side effect that is induced in weight gain. Contrary to this, both cocaine and amphetamine reduce appetite. But, cocaine acts as a potent inhibitor of catecholamine reuptake, but it does not act as an antidepressant. However, excessive cortisol secretion causes depression and is associated with diminished noradrenergic inhibition of corticotropin-releasing hormone secretion in the hypothalamus which induces anxiety in experimental animals. Similarly, triple reuptake inhibitors (TRIs) inhibit serotonin-norepinephrine-dopamine reuptake, enhance monoaminergic neurotransmission by blocking the action of the monoamine transporters, and raise extracellular concentrations of these neurotransmitters in baboons and humans (Table 4) [92].

Similarly, it is also well confirmed that catecholaminergic and serotonergic neurotransmitter systems are implicated in the pathophysiology of attention-deficit/hyperactivity disorder (ADHD). The amino acid tyrosine is the precursor for synthesis of the catecholamines dopamine and norepinephrine, while tryptophan is the precursor of serotonin. Similarly, a disturbed transport of tyrosine, as well as other amino acids, generates number of psychiatric disorders, such as schizophrenia and bipolar disorders. Moreover, an altered tryptophan and alanine transport in fibroblasts causes attention-deficit/hyperactivity disorder in growing juveniles (ADHD [107]. In addition, natural antioxidant compounds, which pass through BBB, are better neuroprotective agents and show novel approach towards excitotoxicity protection and oxidative stress associated with excess *β* amyloid (A*β*) preservation in AD. This is represented by selective glutamatergic antagonists that possess antioxidant capabilities. Both GSH and (R)-α-lipoic acid (LA) get covalently linked with the NMDA receptor antagonists memantine (MEM) and are used in treatments of Alzheimer's disease (AD). It is a symptomatic approach based on the use of cholinesterase inhibitors or N-methyl-D-aspartate (NMDA) receptor antagonists [108]. Norepinephrine transporter (NET) plays important role in pathophysiology of many neurodegenerative diseases such as Alzheimer's disease and hyperactivity disorders [93]. Dopamine, norepinephrine, and serotonin excess and their respective amino acid precursors have also been associated with brain dysfunction [109]. Furthermore, decreased availability of acetylcholine during critical illness leads to decreased counter-regulatory activity in response to inflammatory disease states that causes additional injury and neurotransmitter imbalances (Table 4).

*Serotonin*. Serotonin is a neurotransmitter which is independently synthesized from tryptophan transported across the blood brain barrier. Normally, its 1-2% concentration is found in the brain but highest concentration occurs in the pineal body. Serotonin neurotransmitter neurons are located in the raphe nuclei. Vitamin D hormone regulates serotonin synthesis [110]. Serotonin synthesis is a 2-step process, the first step of which requires the enzyme tryptophan hydroxylase with oxygen, iron, and THB as cofactors. Neither the enzyme nor the cofactors are rate limiting for either step of these reactions. Serotonin concentration in the brain is far more sensitive to the effects of diet than any other monoamine neurotransmitter. Serotonin concentration can be increased up to 10-fold by dietary supplementation in laboratory animals. Hence, consumption of a meal rich in carbohydrate, branch-chained amino acids, and tryptophan has a particularly dramatic effect because both glucose from carbohydrate and branch-chained amino acids (especially leucine) increase insulin secretion. Insulin facilitates the transport of the branch-chained amino acids into muscle cells, thereby reducing the competition tryptophan faces for the large neutral amino acid transporter that takes it across the blood brain barrier. In mammals, noradrenergic neurons near the optic nerve are inhibited by light. In darkness, norepinephrine stimulation of pineal cells causes the release of cyclic AMP second-messenger, which activates (phosphorylates) the N-acetyl transferase enzyme which catalyzes acetylation of serotonin (Table 4).

*Melatonin*. Melatonin (MEL) is an endogenous neurohormone that performs many biological functions and shows powerful antioxidant effect [98]. It is synthesized from serotonin in a 2-step process that takes an acetyl group from acetyl-CoA and a methyl group from by SAMe (s−adenosyl methionine). Melatonin induces pigment lightening in cells and acts as a neurotransmitter that regulates diurnal (circadian) and seasonal behavior and physiology in mammals. Melatonin ameliorates neural function by promoting endogenous neurogenesis through the MT2 melatonin receptor in ischemic-stroke mice [111]. Melatonin shows protective effects against ischemic stroke and reduces oxidative/inflammatory stress. This results in preservation of BBB integrity and enhances endogenous neurogenesis by upregulating neurodevelopmental gene/protein expression [111]. Circulating melatonin can modulate baroreceptor reflex control of HR, thus resetting it toward lower HR values. The modulatory effects of melatonin may be mediated via melatonin receptors in the area postrema, located outside the blood brain barrier (Table 4) [8]. MEL protects the brain tissue from the oxidative stress induced hypobaric hypoxia (HH) and prohibit loss of pericellular haloes, shrunken neurons with scanty cytoplasm and hyperchromatic, pyknotic or absent nuclei; reactive gliosis and edema. Melatonin counteracts the deleterious effects of oxidative stress and defies neuronal death, reactive astrogliosis, memory impairment, and cognitive dysfunctions. Therefore, all dietary supplements containing melatonin are proved highly useful because of their neuroprotective activities for the therapy of hypoxia-induced consequences. Melatonin in humans acts as an inhibitor of sexual activity and also stimulates production of brown adipose tissue, a special form of fat which (when burned) only produces heat, not ATP. This is especially important for hibernating animals (Table 4).

Similar to melatonin, insulin in the brain also regulates the metabolism, molecular composition, and cognitive performance of microcircuits and reduces food intake; cerebral insulin levels are altered in diabetes, aging, obesity, and Alzheimer's disease [112]. Insulin level in the brain is several folds higher than blood plasma levels. Local application of glucose or glibenclamide to neurogliaform cells mimics the excitation suppressing effect of external insulin on local microcircuits via insulin receptors. Thus, neurogliaform cells might link GABAergic and insulinergic action in cortical microcircuits [112]. There is an increase in vascular disorders such as hypertension and Alzheimer's disease (AD). There is association between hypertension and an increased risk of developing AD in population and antihypertensive medications in the management of cognitive disorders which improve independent blood pressure lowering effects and lead to better treatment and/or prevention options for AD [113] (Table 4).

#### 3.8.4. Peptide Neurotransmitters

Peptides are the most common neurotransmitters in the hypothalamus. Their complex structure can allow for high receptor specificity. They are all synthesized on ribosomes and are inactivated by hydrolysis at the synapse rather than by reuptake. Peptides are far more potent than other neurotransmitters, requiring only very small amounts to produce a profound effect. Even very minute amounts of somatostatin can inhibit growth hormone release. Opioid peptides include the endorphins, enkephalins, and dynorphins. Enkephalins are frequently found in presynaptic (axoaxonic) synapses. Opiates and enkephalins (or endorphins) inhibit the firing of locus ceruleus neurons. The highest concentration of opioid receptors is found in the sensory, limbic, and hypothalamic regions of the brain and is particularly high in the amygdala and periaqueductal grey area. Opioids tend to be released as slower-acting cotransmitters which modulate the action of the associated neurotransmitter (such as glutamate) which is being released from the same synapse. Although opioids are generally inhibitory, they have an excitatory effect on hippocampal pyramidal neurons mediated by inhibition of GABA release (Table 4).

Neuropeptides play a crucial role in the normal function of the central nervous system and peptide receptors hold great promise as therapeutic targets for the treatment of several CNS disorders[104]. But, it is very difficult to harness drug-like properties of peptides to transform them into marketable therapeutic molecules because of their poor stability, solubility, and incompatibility. Moreover, recent technical advances have broken these limitations and so many therapeutic peptide drugs are developed to treat a wide range of diseases such as diabetes, cancer, and pain. Further, the same is favored and practiced by clinicians for potential utility of agonists at central neurotensin, cholecystokinin, neuropeptide Y, and oxytocin receptors [104]. Moreover, successful approaches have been developed to increase the stability and longevity of peptides* in vivo* (Table 4). It leads to the improvement in delivery because of easy penetration across the blood brain barrier, [104]. Further, therapeutic potential of peptide agonists is also harnessed for the treatment of major CNS disorders such as schizophrenia, anxiety, depression, and autism.

Neuropeptides are signaling molecules which participate in the modulation of synaptic transmission and are stored in dense core synaptic vesicles. These are released after profound excitation [100]. Only in the extracellular space, neuropeptides act on G-protein coupled receptors to exert a relatively slow action both pre- and postsynaptically [100].Therefore, neuropeptide modulators are considered ideal candidates to influence epileptic tissue overexcited during seizures. Many of these peptides are considered to participate in endogenous neuroprotective actions because they bind to their own receptors that implicate them in epilepsy and other CNS disorders. Mainly, neuropeptide receptors occur in the hippocampus and are widely concerned to temporal lobe epilepsy. Similarly, receptors of other neuropeptides like somatostatin, neuropeptide Y, galanin, dynorphin, enkephalin, substance P, cholecystokinin, vasoactive intestinal polypeptide, hormones such as ghrelin, angiotensins, corticotropin-releasing hormone, adrenocorticotropin, thyrotropin-releasing hormone, oxytocin, and vasopressin involved in epilepsy [100]. Therefore, activation and inhibition of receptors by oral application of peptides as drugs are typically not efficient because of low bioavailability, rapid degradation, and insufficient penetration of peptides through the blood brain barrier. Further, development of nonpeptide agonists and antagonists of neuropeptide receptors as well as gene therapeutic approaches leads to the local production of agonists and antagonists within the central nervous system (Table 4) [100].

Similarly, neuropeptide substance P (SP) has been implicated in inflammation, pain, depression, and breast cancer cell (BCC) growth. SP plays role in trafficking of BCCs (human MDA-MB-231 and MDA-MB-231BrM2 cells) across the blood brain barrier (BBB) and brain microvascular endothelial cells (BMECs) using* in vitro* and* in vivo* models [114]. The proinflammatory peptide substance P promotes blood brain barrier breaching by breast cancer cells through changes in microvascular endothelial cell tight junctions. SP secreted from BCCs induces transmigration of BCCs across the BBB, leading to activation of BMECs and secretion of TNF-α and Ang-2, resulting in BBB impairment and colonization of tumor cells in brain (Table 4). Therefore, therapies based on SP inhibition in combination with other therapies may prevent breaching of the BBB by BCCs and their colonization in brain (Table 4) [114].

Similarly, gastrointestinal hormones act in the central regulation of energy metabolism and show potential sensory roles for the circumventricular organs [103]. These generate a variety of circulating signals which provide essential information to the central nervous system (CNS) regarding nutritional status. The gastrointestinal system produces many of such molecules that show profound effects on feeding behavior and the control of metabolism as a consequence of their ability to regulate the neural circuitry involved in metabolic homeostasis. Many of these substances show lipophobic characteristics which could be used for therapeutics diseases neurosensory organs [103]. More often, sensory circumventricular organs (CVOs), found in the brain, are not protected by the normal blood brain barrier. These possess receptors for and functional actions of gastrointestinal hormones such as amylin, cholecystokinin, ghrelin, and peptide YY in the area postrema and subfornical organ. These play significant roles for the sensory CVOs in the regulation of energy balance (Table 4) [103].

Cholecystokinin (CCK) seems to function in the production of satiety; administration of small quantities of this peptide into the ventricles or the paraventricular nucleus inhibits feeding. It is associated with dopamine synapses in some limbic areas and appears to modulate dopamine release. Such peptide synergy with other transmitters is common. These neuropeptides show synergy as GABA is found associated with somatostatin and serotonin with substance P. Similarly, low doses of the peptide vasopressin can enhance learning in laboratory animals, but humans with vasopressin deficiency show no signs of memory impairment. It increases blood pressure and should be approached with caution. Safer analogues may yet be found. Similarly, leptin regulates energy expenditure and body weight by acting both on the hypothalamus and on peripheral targets [101] and central actions of leptin are enhanced by cholecystokinin (CCK). The interaction between leptin and CCK causes physiological alterations in animals experimental after infusion of more leptin into the CNS [101]. Similarly, apolipoprotein AIV (apo-AIV) and cholecystokinin (CCK) concentration generate gastrointestinal satiation signals that are stimulated by fat consumption [102].Therefore, peripheral apo-AIV requires an intact CCK system and vagal afferents to activate neurons in the hindbrain to reduce food intake [102].

#### 3.8.5. Biogenic Amine Neurotransmitters

The trafficking of neurotransmitters within the brain depends greatly on the so-called uptake1 and uptake2 carrier-mediated processes, which maintain the balance between neurotransmitters in the intracellular and extracellular fluids of the nervous parenchyma [68]. These transporters also make the extracellular clearance of biogenic amine neurotransmitters at synaptic clefts. Both uptake systems regulate the neurotransmitter concentration and modulate/terminate receptor-mediated effects within the neurovascular unit (NVU). Uptake2 (Oct1-3/Slc22a1-3, Pmat/Slc29a4) and Mate1/Slc47a1 transporters are also involved in the transport of xenobiotics. More specifically, functional Net and Oct1 transporters occur at the luminal BRB surface. These heterogeneous transport properties of the brain and retina NVUs suggest that the BBB helps protect the brain against biogenic amine neurotransmitters in the plasma, while the BRB has more of a metabolic/endocrine role (Table 4) [68].

More specifically, both transporters uptake1 and uptake2 play important role in the clearance of dopamine, serotonin, and norepinephrine at the synaptic cleft [94, 97]. These are classified based on their affinity/capacity for substrates/inhibitors and sensitivity to ions. More specifically, uptake1 transporter binds with high-affinity to Na^+^/Cl^−^-dependent unidirectional symporters such as Net (Slc6a2), Dat (Slc6a3), and Sert (Slc6a4), while uptake2 transporters show lower affinity. Uptake1 is polyspecific and shows binding to Na^+^/Cl^−^-independent symporters. Uptake1 transporter assists in transportation of some biogenic amine neurotransmitters and xenobiotics in both directions that depends on their concentration gradient. Among all symporters, best known are Oct1-3 (Slc22a1-3) and Pmat [95, 96, 115]. It is also true that trafficking of biogenic amine neurotransmitters is not restricted to neurons but it might also occur at the blood-nerve barriers and in some peripheral tissues [116–118]. Therefore, it is much possible that both uptake1/2 transporters could modulate the local availability of biogenic amine neurotransmitters by luminal (blood) and/or abluminal (nerve side) carrier systems. More exceptionally, uptake2 carriers could also transport many drugs and xenobiotics like 1-methyl-4-phenylpyridinium (MPP^+^) (Table 4) [69].

### 3.9. Exchange of Metal Ions between Plasma and Brain

Capillaries in the brain possess an impermeable barrier to some solutes and regulate the movement of substances between blood and brain. These brain capillary endothelial cells facilitate the transcapillary exchange of some salts. Moreover, for sodium and potassium transport, endothelial cell contains distinct types of ion transport systems on the two sides of the capillary wall, that is, the luminal and antiluminal membranes of the endothelial cell (Table 5) [119]. Therefore, highly specific solutes can be pumped across the capillary against an electrochemical gradient. These transport systems play important role in the active secretion of fluid from blood to brain and in maintaining a constant concentration of ions in the brain's interstitial fluid [120]. Further, to perform major physiological functions, exchange of important metal ions such as K^+^ and Na^+^ takes place very fast from blood to tissues, but it shows slow rate when entered into the brain [41, 121]. More specifically, Na^+^ exchange across the blood brain barrier takes place by carrier-mediated transport [120], mainly by brain capillary Na^+^, K^+^ATPase system [122]. This is located primarily on the antiluminal membrane of the endothelial cell that may also mediate removal of interstitial fluid K^+^ from the brain and maintain a constant brain K^+^ concentration in the face of fluctuating plasma concentrations. Thus, antiluminal location of Na^+^, K^+^-ATPase may have important role in secretion of interstitial fluid, an extrachoroidal source of CSF (Table 5) [119].

More specifically, plasma membrane of metazoan cells possesses multiple transport proteins, Na^+^/K^+^ pump, K^+^channel, Na^+^ lysine symporter channel, and sodium-lysine transporter which transport metal ions and amino acids from the extracellular medium into the cell. These transporter proteins facilitate active movement of small molecules or ions [34]. There occur three different types of transport proteins; first type is the uniport transport pass, a single type of molecule down its concentration gradient, while symporters are cotransport proteins which allow the movement of both types of ions in the same direction outside to inside. An antiporter allows movement of one molecule against its concentration gradient and never allows other molecules to go inside. More specifically, pumps utilize the energy released by ATP hydrolyzed by Na^+^K^+^ATPase for active transport of specific ions or small molecules against their chemical gradient. Contrarily, these channels permit movement of specific ions or water down their electrochemical gradient. Third type of ionic movement is performed by membrane transporter proteins such as ABC transport proteins (Table 5) [35].

Further, it is also true that brain lacks some metal transporters, and blood brain barrier stops excess transport of metallic ions. BBB restricts entry of neurotoxic metals into the central nervous system (CNS) that may cause neurodegenerative diseases. Though it is a hard fact that excess of metals (Cu, Fe, Mn, and Zn) contributes to neurodegenerative diseases, but in trace amount these are essential for human health [33]. Pb, tin, and Hg requirement is not very clear; hence, its transport occurs very scarely. Moreover, plasma membrane of rat brain neuronal cells also contains zinc transporters (Table 5) [33].

But, aluminium uptake takes place by transferrin-receptor-mediated endocytosis [36]. Few hydrophilic proteins like transferrin carry metal species, such as the free metal ion and complexes of the metal with an amino acid or protein. But, these would not be expected to be able to distribute metal ions across the blood brain barrier (BBB) at a rate that is sufficient to meet the requirements of the brain as BBB limits the diffusion of non-lipophilic substances into brain. An example of metal species specific transport at the BBB involves Me Hg, which forms a complex with L-cysteine that is transported into the brain and perhaps into and out of astrocytes. Cu is delivered to the CNS but it must be transported across either the blood brain barrier or the CSF barrier and it accumulates within brain capillaries of experimental mice [28–30]. ATP 7A catalyzes the stepwise transport of Cu across brain endothelial cells and astrocytes to supply neurons with a regulated supply of Cu [31, 32] that is quite similar of brain Fe metabolism [130]. Fe uptake occurs across the cerebelar vascular endothelium after which it associates with glial cells and neurons for take-up. Choroid plexus actively produces and secretes CSF and has pivotal role in maintaining the homeostasis, but it may also provide a pathway for metal transport mainly for Cu. [131]. Androstane causes receptor-mediated upregulation of ATP-driven xenobiotic efflux transporters at the blood brain barrier [35], while blood brain barrier flux of aluminum, manganese, iron, and other metals is suspected to contribute to metal-induced neurodegeneration (Tables 1 and 5) [36].

Certain metal transporters maintain metal flux across the blood brain barrier, choroid plexuses as well as in sensory nerves, and this metal uptake occurs from the nasal cavity [37]. Thus, for maintaining homeostasis and normal body health, physiological amounts of useful metals are transported by protein transporters. But, blood brain barrier flux of aluminum, manganese, iron, and other metals is suspected to contribute to metal-induced neurodegeneration and causes dyshomeostasis [36]. Aluminium uptake takes place by transferrin-receptor-mediated endocytosis and of aluminum citrate by system Xc and an organic anion transporter [132]. Contrary to this, manganese uptake takes place by transferrin- and non-transferrin-dependent mechanisms, which may include store-operated calcium channels and the lack of transporter-mediated manganese brain efflux. Iron uptake takes place by both transferrin-dependent and independent mechanisms of brain, while copper is delivered to the brain by copper transporters, ATP7A, and ATP7B. Membrane transporters are often quite substrate specific. For example, the divalent metal transporter (DMT1, DCT1, and Nramp2) transports divalent metals, but not metals in other valence states. Another example of substrate specificity is greater binding affinity of transferrin for Fe^+3^ than Fe^+2^ [36, 38, 39] (Tables 1 and 5). As a result, transferrin-receptor-mediated endocytosis (TfR-ME) for Fe^+3^ occurs more than for Fe^+2^ [36, 38, 39]. Furthermore, the affinity of the transferring receptor for halo transferrin (diferric transferrin) is considerably greater than for monoferric transferring [42]. TfR-ME is also believed to play a role in the transport of other trivalent metals into the brain, such as Al and Mn. Similarly, there seems to be a putative role of zinc transporters, ZnT and Zip, in regulating brain zinc concentration by uptake mechanisms. Zinc uptake is facilitated by L- and D-histidine. Brain uptake of metals may involve a non-energy-dependent process, store-operated calcium channels, and/or an ATP-dependent calcium pump [36]. Methyl mercury can form a complex with L-cysteine that mimics methionine, enabling its transport by the L system (Tables 1 and 5) [43].

The transport of essential metals and other nutrients across tight membrane barriers such as the gastrointestinal tract and blood brain barrier is mediated by specific transport mechanisms [133]. These transporters take up metals at the apical surface and export them at the basolateral surface and are involved in their intracellular distribution. Transporters for each of the major essential metals, calcium, iron, and zinc are separate and are divalent metal transporter 1 that mediate the transport of nonessential metals across tight membrane barriers. For example, the intestinal iron transporter divalent metal transporter 1 mediates the uptake of lead and cadmium. Thus, levels of essential metals are strictly regulated by transporters [133]. When dietary levels of essential metals become low, levels of the corresponding transporters increase in the intestine, after which there is a greater potential for increased transport of toxic metals. In the brain, the strict regulation of metals prevents injury that would potentially result from oxidative damage induced by the essential metals iron, copper, and zinc. Indeed, the oxidative damage found in neurodegenerative diseases is likely to be due to higher levels of these metals [133]. Involvement of intracellular transporters raises the levels of iron, zinc, and copper that might be due to a disruption in the activity of transporters in Alzheimer's disease. Similarly, exposure to toxicants also affects the activity of transporters that potentially could contribute to the aetiology/progression of neurodegenerative diseases (Tables 1 and 5) [133].

The regulation of metal ion transport [120] within neurons is critical for normal brain function [40]. Moreover, for regulation of redox, metals such as iron become essential (Fe), because excess levels of these metals can contribute to oxidative stress and protein aggregation leading to neuronal death. More importantly, divalent metal transporter 1 (DMT1) plays a central role in the regulation of Fe as well as other metals; hence, failure of DMT1 regulation is linked to human brain pathology. DMT1 is regulated by Ndfip1 (Nedd4 family-interacting protein 1) an adaptor protein that recruits E3 ligases to ubiquitinate target proteins. In human neurons, Ndfip1 is upregulated and binds to DMT1 in response to Fe and cobalt (Co) exposure [40]. This interaction results in the ubiquitination and degradation of DMT1, resulting in reduced metal entry, while induction of Ndfip1 expression protects neurons from metal toxicity, and removal of Ndfip1 by shRNAi results in hypersensitivity to metals. More specifically, Nedd4-2 as an E3 ligase is recruited by Ndfip1 for the ubiquitination of DMT1 within human neurons and Ndfip1(−/−) brains accumulate Fe within neurons and suggest a critical role for Ndfip1 in regulating metal transport in human neurons (Tables 1 and 5) [40].

DMT1 possesses four or more isoforms that transport eight different metals, for multiple purposes [134]. Two mRNA isoforms differ in the 3′ UTR; +IRE DMT1 has IRE (iron responsive element) but −IRE DMT1 lacks this feature. The +/−IRE proteins differ in the distal 18 or 25 amino acid residues after shared identity for the proximal 543 residues. The +/−IRE proteins as the apical iron transporter in the lumen specifically, −IRE form participate in metal detoxification and remove out metal toxicity. It plays important role in management of toxic challenges and metal homeostasis in body tissues [134] and is involved in endosomal exit of iron. Normally, airways represent a gateway tissue for metal entry.

Furthermore, divalent metal-ion transporter 1 (DMT1) is iron-preferring membrane transport protein that plays indispensable roles in intestinal nonheme-iron absorption and iron acquisition by erythroid precursor cells. Rare mutations in human DMT1 result in severe microcytic-hypochromic anemia. An iron responsive element (IRE) in the mRNA 3′-untranslated region permits the regulation of some isoforms by iron status [135]. More specifically, natural resistance associated with macrophage protein 1 (NRAMP1) is the only member of the mammalian SLC11 gene family which contributes to antimicrobial function by extruding from the phagolysosome divalent metal ions (e.g., Mn^2+^). SLC11 gene also encodes proteins like divalent cation transporter 1 (DCT1) and solute carrier family 11 [136, 137]. Possibly, it may be essential cofactors for bacteria-derived enzymes or required for bacterial growth. An intestinal nonheme-iron transporter, DMT1, is a validated therapeutic target in hereditary hemochromatosis (HHC) and other iron-overload disorders [135]. DMT1 represents a large family of orthologous metal ion transporter proteins that are highly conserved from bacteria to humans [138]. As its name suggests, DMT1 binds a variety of divalent metals including cadmium (Cd^2+^) and copper (Cu^2+^). DMT1 is best known for its role in transporting ferrous iron (Fe^2+^) and its expression is regulated by body iron stores to maintain iron homeostasis (Tables 1 and 5). DMT1 is also important in the absorption and transport of manganese (Mn^2+^) [139]. More specifically, during brain injury, the homeostasis of transition metal ions (e.g., Fe^2+^, Co_2_^+^) is grossly unbalanced. This renders the brain environment toxic due to unwelcomed and uncontrolled metal entry into stressed neurons, precipitating in their premature death. Ndfip1 protects neurons against excess transition metal exposure. In the presence of excess Co_2_^+^ or Fe^2+^ ions, Ndfip1 is upregulated. Moreover, Ndfip1 binds to DMT1, leading to DMT1 ubiquitination and degradation, inhibits metal entry, and allows neuronal recovery. Ndfip1 has larger relation to brain diseases (e.g., Parkinson's disease) arising from metal accumulation. Ndfip1 protects human neurons from death by ubiquitination and degradation of metal transporter DMT1.DMT1 may be the major transporter of manganese across the blood brain barrier and expression of this protein in the nasal epithelium provides a route for direct absorption of metals into the brain [140].

DMT1 expression is found to be increased in the substantia nigra of Parkinson's patients and in the ventral mesencephalon of animal models. Its expression in the brain increases with the age [141] that also increases the susceptibility to metal induced pathologies. Moreover, the DMT1 encoding gene SLC11A2 occurs on the long arm of chromosome 12 (12q13) close to susceptibility regions for Alzheimer's disease [142] and restless legs syndrome. Similarly, C allele of SNP rs407135 on the DMT1 encoding gene SLC11A2 is associated with shorter disease duration in cases of spinal onset amyotrophic lateral sclerosis [143]. DMT1 is also found implicated in Alzheimer's disease onset in males as well [142]. Moreover, CC haplotype for SNPs 1254T/C IVS34+44C/A is associated with Parkinson's disease susceptibility [144], while variant alleles on several SLC11A2 SNPs are associated with iron anemia (Tables 1 and 5). These are also found as a risk factor for manganese intoxication and restless legs syndrome [145]. Therefore, it is true that the lack or overexpression of transporters causes toxic accumulation of divalent metals, especially iron and/or manganese. It generates metal deficiency and enhances cellular responses in affected persons [134]. Moreover, lack of metal transporters works as important aetiological factors in a variety of neurodegenerative diseases, including Alzheimer's disease, Parkinson's disease, amyotrophic lateral sclerosis, and multiple sclerosis.

### 3.10. Transport of Amino Acids

Brain capillary endothelial cells are connected by extensive tight junctions and are polarized into luminal (blood-facing) and abluminal (brain-facing) plasma membrane domains. This affects polar distribution of transport proteins and mediates amino acid (AA) homeostasis in the brain. The existence of two facilitative transporters for neutral amino acids (NAAs) on both membranes provides the brain access to essential AAs. More specifically, there occurs a common carrier/transport system for large amino acids across the blood brain barrier which regulates concentration of amino acid and takes out excess or elevated level of amino acids. More often, carrier/transport system can lower down the transport of amino acids by competitive inhibition or may convert into the required amino acids. But, distinct transport systems facilitate the uptake of basic, acidic, and beta amino acids. In case of phenylketonuria and maple syrup urine disease, few amino acids like phenylalanine accumulated in excess in the blood stop uptake of other essential amino acids. Further, excess of amino acids affects the LNAA carrier and allows transfer of more amino acids beyond damaging level into the CNS. Thus, large amino acids like phenylalanine, leucine, tyrosine, isoleucine, valine, tryptophan, methionine, and histidine enter the cerebrospinal fluid rapidly like glucose through the common carrier system or a single amino acid transporter system. Possibly, it may be a leucine preferring transporter. On the other hand, entry of small amino acid such as alanine, glycine, proline, and *γ*-aminobutyric acid (GABA) is restricted or controlled because higher influx of these amino acids may enhance the control of neurotransmitters. These amino acids are required for syntheses of neurotransmitters in the brain and are selectively transported out of the CNS into the blood through amino acid carrier system or alanine-preferring transporter and 1-dihydroxy-phenylalanine (1-DOPA). In addition, few smaller neutral amino acids synthesized in the brain and several of them act as putative neurotransmitters. But, all these neutral L-amino acids show variable transport into the brain by different transport systems [123, 124]. Neutral L-amino acids transport depends on amino acid concentration and its utilization because excess amount of it may stop the transport. More specifically, amino acid that cannot be synthesized by brain and are metabolically essential require its regular supply. It is maintained by protein breakdown and dietary supply of amino acids. Contrarily, both transport and uptake of large amino acids into the brain are inhibited by the synthesis of 2 aminonorbornane-2-carboxylic acids but not by 2-(methyl-isobutyric acid (MeAIB) [123, 124]. This is the main reason that supply of neutral amino acids, such as alanine, glycine, proline, and *γ*-aminobutyric acid (GABA), is restricted into the brain by these inhibitors [125]. Some of these neutral large amino acids are transported by A-(alanine-preferring)-system carrier [64]. This carrier system is not found on the luminal surface of the blood brain barrier but may occur on antiluminal surface of the brain capillary [126]. There is a contrast in its transportation because distinct transport systems facilitate the brain uptake of basic, acidic, and *β*-amino acids. Lysine and arginine are essential amino acids which are provided from the blood after dietary assimilation. The acidic amino acids glutamate and aspartate are both important metabolic intermediates as well as neurotransmitters [127, 128], while the brain content of these amino acids is maintained primarily by* de novo* synthesis. They also can be transported into the brain at a slow rate across the blood brain barrier. The *β*-amino acid taurine is present at high concentrations in the brain and is involved in volume regulation. Absorption surface in GI tract contains brush boarder membrane that possess a novel system which mediate Na^+^/neutral amino acids [146] and show Na^+^-gradient-dependent transport of L-proline [129]. More specifically, transport of cationic and zwitterionic amino acids [147] and methyl mercury transport across the blood brain barrier occurs by an amino acid carrier [148]. Both leucine and alanine are taken up by cultured cerebral capillary endothelial cells [149].

### 3.11. Transport of Proteins

Delivery of therapeutic peptides and proteins to the central nervous system is the biggest challenge for development of more effective neuropharmaceuticals [105]. Many plasma proteins are not able to cross the endothelial tissue barrier because of their large size and hydrophilicity. This is the main reason why concentrations of certain proteins, such as insulin and transferrin, vary with the change in plasma concentrations. Consequently, their concentrations in the brain become very low except few exceptions. Due to controlled uptake of some proteins, brain becomes saturable and their further uptake is stopped. Receptor-mediated transcytosis across the blood brain barrier may be useful way to transport protein and peptide therapeutics into the brain [150]. Moreover, proteins like insulin, transferrin, insulin-like growth factors, and vasopressin cross the BBB by receptor-mediated transcytosis (Table 4) [123]. Interestingly, protein receptors occur in outnumber in brain capillary endothelial cells which bind to transporting protein and form a receptor complex. This complex is endocytosed into the endothelial cell to form a vesicle which assists in eventual release of intact protein on the other side of the endothelial cells that is still not fully known. Contrary to this, blood brain barrier is impermeable to most molecules [105] and only amphiphilic derivative of a peptide could make it possible to deliver into the brain. For this purpose, a drug peptide is suitable and designed to self-assemble into nanofiber and its active epitope is tightly wrapped around the nanofiber core [105]. Thus, a novel drug delivery system could maintain high brain permeability through receptor-mediated endocytosis (Table 5).

Moreover, mechanism of nanoparticle mediated drug transport across the BBB appears to be receptor-mediated endocytosis [151]. Thus, transport of nanoparticles/nanomolecules across the blood brain barrier requires both specific and nonspecific interactions with proteins expressed on the luminal and or abluminal surface of the brain endothelium cells [152]. More specifically, a high-affinity iron chelator is conjugated to lactoferrin molecules by carbon dioxide mediated coupling reaction [153]. Similarly, Tf-FMMs transferring-conjugated fluorescein loaded magnetic nanoparticles are also delivered by receptor-mediated transcytosis [63], while antitransferrin receptor antibodies with a Fab cargo are delivered across the BBB by receptor-mediated endocytosis [154]. In another method, polycationic proteins and lectins are allowed to cross the blood brain barrier by a similar but nonspecific process called absorptive-mediated transcytosis. In this process, peptide binding to specific receptors in the membrane is not mandatory and proteins are directly absorbed to the endothelial cell membrane based on charge or affinity for sugar moieties of membrane glycoproteins. Rests of the subsequent transcytotic events are followed similar to receptor-mediated transcytosis. It is also important that transport capacity of absorptive-mediated transcytosis is greater because the number of receptors present in the membrane does not limit it. In addition, cationization may provide a mechanism for enhancing brain uptake of almost any protein but both endocytosis and transcytosis mechanisms play important role in the distribution of macromolecules [152]. The degree of passage of small peptides across the blood brain barrier significantly exceeds that of the vascular markers, while peptide transport across the membrane barriers shows either saturable or facilitated transport and* transmembrane diffusion* or passive diffusion. More specifically, low molecular weight small peptides are transported* in* and* out *of the central nervous system by carrier-mediated transcytosis across the blood brain barrier which acts as a saturable mechanism (Table 4).

### 3.12. Transport of Drugs

BBB is impermeable to proteins, drugs, and other biomolecules as these are not able to cross the blood brain barrier because of their size and hydrophilicity. But, few peptide hormones which regulate body metabolism and normal functions are transported easily. Similarly, both insulin and transferring across BBB and their concentration vary in plasma because its uptake in the brain is greater due to their size and lipid solubility. These are carried to the brain by specific transport processes mainly membrane bound efflux pumps and channels. The major transport mechanism which carries important proteins and hormones is receptor-mediated transcytosis. Contrary to this, BBB restricts entry of most of the biomolecules mainly proteins, peptides, carbohydrates, and vaccines. Hence, delivery of therapeutic peptides and proteins to the central nervous system is the biggest challenge for development of more effective neuropharmaceuticals [105]. More often, therapeutic agents may reach the target sites at intracellular locations. The brain capillary endothelial cell is highly enriched in receptors for these proteins, and following binding of protein to the receptor, a portion of the membrane containing the protein-receptor complex is endocytosed into the endothelial cell to form a vesicle. Although the subsequent route of passage of the protein through the endothelial cell is not known, there is eventual release of intact protein on the other side of the endothelial cell. The blood brain barrier is impermeable to most molecules [105] but during trafficking of biomolecules a portion of compound is lost due to ineffective partitioning across the membrane. This partitioning across the membrane is widely concerned to polarity, lipophilicity of molecules that attribute easy passage across the membrane. Therefore, amphiphilic derivatives of a peptide are easily delivered into the brain. It was designed to self-assemble into nanofiber which in the active peptide epitope is tightly wrapped around the nanofiber core [105].

Recently, several neuroprotective proteins and peptides of potential therapeutic value have been designed and used to fulfill the crucial need for effective and safe transcapillary delivery methods to the brain. Hence, most promising drug delivery through brain capillaries is possible by augmentation of pinocytotic vesicles. This is a cellular mechanism in which large molecules of neurotherapeutic potential are conjugated to peptidomimetic ligands for delivery. Later on, these molecules bind to selected peptide receptors, which internalize and transported it in small vesicles across the cytoplasmic brain capillary barrier. These conjugates were found functionally active and effective in animal models of neurological disease. Similarly, neurotrophin, a brain-derived neurotrophic factor, easily passes through BBB and has great therapeutic value. Interestingly, all short peptides with hydrophilic nature showed favorable safety profiles in brain after coming across the BBB found neuroprotective. Exogenous recombinant human erythropoietin was proved beneficial in treating global and focal cerebral ischemia and reducing nervous system inflammation in experimental animals. Moreover, other than neuroprotective compounds monoclonal antibodies are also used to pass through BBB by receptor-mediated endocytosis mechanism. Similarly, metallothioneins a superfamily of highly conserved amino acids contains low molecular weight polypeptides play significant role in the regulation of essential metals which are also internalized by receptor mediated endocytosis. Thus, variable efficiencies of endocytosis mechanisms, such as intracellular trafficking, aiding in the release of therapeutic agents into the cytoplasm are important aspects in drug delivery and therapeutic potency. There are possibilities that after diffusion and translocation of the therapeutic agents these remain susceptible targets of certain catalytic enzymes or physically partition into the nucleus or in any other suborganelles that may also alter its actual activity. Further, excess delivery of therapeutic agents may create competitive problem to some other biomolecules that may hinder normal functions of cells, cellular organelles, enzymes, and signaling molecules as well. In addition, metabolic wastes may also overburden the cell cytoplasm that inhibit so many normal cellular functions and give rise to drug induced adverse effects. Use of nanoparticles may solve this problem due to controlled release of drugs in required quantity. These can easily cut down concentration of metabolic waste materials by masking the therapeutic agents from their biological environment. Nanoparticles allow controlled (sustained) drug release from the matrix, determine required bioavailability, and show reduction of the dosing frequency. These are proved the most successful drug carriers due to their high stability, high carrier capacity, and feasibility of incorporation of both hydrophilic and hydrophobic substances into brain or inside cells. These also show feasibility to deliver drugs by following variable routes of administration, including oral application and inhalation.

Normally, two mechanisms are employed to ascertain that the internalization of biomolecules mainly liquids is liquids are seep in by pinocytosis and solids by phagocytosis. But, carrier-mediated delivery of drugs by nanoparticles is also developed in which nanoparticles are ingested by cells from the medium or from any microenvironment surrounding the cell. More often, nanoparticles are pouring in by receptor-mediated endocytosis that operates by membrane manipulation to envelope and allows materials to absorb inside. Moreover, nanoparticles get inside the cells by three different mechanisms, that is, phagocytosis, pinocytosis, and receptor-mediated endocytosis. Phagocytosis is associated with few cells types such as macrophages, neutrophils, and dendritic cells that can absorb materials of micrometer in size, that is, 10 *μ*m in diameter. Similarly, pinocytosis is a universal mechanism which occurs in all cell types and it delivers different types of liquids having a submicron size and substances in solution inside the cell. More specifically, larger sized nanoparticles are taken up by the cell by phagocytosis, while smaller ones are absorbed by pinocytosis and almost ingested by all cell types. Therefore, both types of nanoparticles have important but separate advantages. Furthermore, there is another mechanism which is known as absorptive-mediated transcytosis that is especially used to traverse polycationic proteins and lectins. This is a nonspecific process in which biomaterials rather than binding to specific receptors in the membrane, proteins absorb the endothelial cell membrane based on charge or affinity for sugar moieties of membrane glycoproteins. Its subsequent transcytotic events are probably similar to receptor-mediated transcytosis. Hence, overall capacity of absorptive-mediated transcytosis is far greater than that of receptor-mediated endocytosis because the number of receptors present in the membrane does not limit it. Thus, cationization may provide a mechanism for enhancing brain uptake of almost any protein.

In case of facilitated diffusion (a form of carrier-mediated endocytosis) binding of a ligand occur to a transporter on one side of the membrane that triggers a conformational change in the protein receptor. Facilitated diffusion is passive (i.e., energy independent) and contributes to transport at the BBB of substances such as monocarboxylates, hexoses amines, amino acids, nucleoside, glutathione, and small peptide. [155]. Carrier-mediated transport can also be divided into a number of different mechanisms dependent on energy and/or cotransport of another substance. Cotransport may be in the same direction (symport) or in the opposite direction (antiport). This process proceeds from a region of high concentration to a region of low concentration. Endocytosis can be segregated into bulk-phase, also known as fluid phase, endocytosis, and mediated endocytosis (receptor and absorptive-mediated). Bulk-phase endocytosis (pinocytosis) is the nonspecific uptake of extracellular fluids and occurs at a constitutive level within the cell via mechanisms, which are independent of ligand binding [156]. Bulk-phase endocytosis is temperature and energy dependent, noncompetitive, and nonsaturable. Bulk-phase endocytosis occurs to a very limited degree in the endothelial cells of the cerebral microvasculature [157].

Receptor-mediated endocytosis (RME) provides a means for selective uptake of macromolecules. Cells have receptors for the uptake of many different types of ligands, including hormones, growth factors, enzymes, and plasma proteins. RME occurs at the brain for substances, such as transferrin [158], insulin [159], leptin [160], and IGF-I and IGF-II [161], and is a highly specific type of energy dependent transport. Substances that enter a cell by means of RME become bound to receptors that collect in specialized areas of the plasma membrane known as coated pits. The coated pits contain the electron dense clathrin protein and other proteins [162]. When these bound to ligand, pits invaginate into the cytoplasm and then pinch free of the plasma membrane form coated vesicles. The clathrin vesicle coat is rapidly removed to form smooth-coated endosomes that form a compartment of uncoupling receptor and ligand (CURL) [163]. The endosomal membrane contains proton ATPases that result in acidification of the endosome interior and dissociation of the ligand from the receptor within the CURL.

Absorptive-mediated transport (AME) is triggered by an electrostatic interaction between a positively charged substance, usually a charge moiety of a peptide, and the negatively charged plasma membrane surface (i.e., glycocalyx) [164]. AME has a lower affinity and higher capacity than receptor-mediated endocytosis. The development of many new drug delivery technologies focuses on AME [165]. Another significant transport mechanism at the BBB is carrier-mediated efflux. This mechanism is involved in extruding drugs from the brain and is a major obstacle for many pharmacological agents. ABC (ATP binding cassette) transporter P-glycoprotein is the principle efflux mechanism of these agents [166]. There also exist efflux transporters for organic anions, via multidrug resistance associated protein (MRP) [167], and anionic and cationic cyclic peptide [168]. Additionally, the peptide transport system- (PTS-) 1 shows efflux transport of synthetic opioid peptide Tyr-MIF-1 [169]. Peptides are the most common neurotransmitters in the hypothalamus. Their complex structure can allow for high receptor specificity. They are all synthesized on ribosomes and are all inactivated by hydrolysis at the synapse (rather than by reuptake). Peptides are far more potent than other neurotransmitters, requiring only very small amounts to produce a profound effect. Even very minute amounts of somatostatin can inhibit growth hormone release.

#### 3.12.1. CNS Protection

Drug delivery to the brain is a challenging task because presence of blood brain barrier (BBB) complicates the delivery of drugs to the brain [64]. It is a physiological barrier which imposes major obstacle for a large number of drugs, including antibiotics, antineoplastic agents, and neuropeptides, to pass through the endothelial capillaries to brain. There are some other reasons; that is, drug may bind to nontransporters in larger amount that render the drug ineffective or seem theoretically active. But, it is true that drug might show inability to pass through the barrier with the adhered protein. Besides, enzyme action may also make the drug inactive or in a nontherapeutic intermediate compound. All these circumstances with the drug and its delivery must be accounted for making effective drug formulations to treat the CNS disease [27]. Though, several methods, ways, and strategies have been developed for drug delivery to the brain but most of them are proved invasive and lack the target specificity. Therefore, for active transfer of drug to the brain, safer disruption of BBB or its loosening is highly important. Further, for successful delivery of drugs to the CNS, blood brain barrier disruption or opening with intra-arterial infusion therapy allows both the chemotherapeutic agents and antibodies to enter through blood brain barrier [20]. Thus, BBB dysfunction could be of great therapeutic value in conditions in which neuronal damage is secondary or exacerbated by BBB damage. More exceptionally, for delivery of drugs or any therapeutic agent, BBB is disrupted by ultrasonic sound waves or it may forcibly break down by sudden raised high blood pressure due to hypertension, trauma, ischemia, neural inflammation, and very high concentration of substances, microwave, radiation, and exposure to infectious agents that can open the BBB. In addition, reducing, halting, or reversing the structure and function of BBB might open new ways to deliver chemotherapeutic agents in case of brain tumor. But, forced opening or structural damage to the BBB allows the uncontrolled passage of drugs [13]. Further, there are several areas of the brain where BBB is very thin or supposed to be loose or weak; from here, drug can allow pass to the brain. This also allows important metabolic substances to cross into the brain somewhat freely. These areas of brain are identified as circumventricular regions; these are pineal body, neurohypophysis, and area postrema.

Further, drug delivery strategies have been improved for safe delivery of different types of drugs to CNS. These improved strategies include liposomes, colloidal drug carriers, micelles, chimeric peptide technology, intranasal and olfactory route of administration, and nanotechnology. Moreover, disruption or damage of endothelium could allow expression of endothelial receptors which are normally downregulated, opening new communication loops between endothelium, pericytes, astrocytes, and microglia. These also play important role in barrier repairing. Moreover, infectious pathogenic viruses affect different tissue and organs, but CNS diseases are severely caused by virus invade various regions of brain. Therefore, drug delivery methods that could target virus present in these areas may play important role in modern drug development mainly for CNS protection. Furthermore, nanoenabled delivery systems have been made that offer more promising solution to enhance the targeted delivery of the drugs into the brain. Therefore, drugs which are loaded with nanocarriers can easily target virus multiplication inside brain because they could easily pass through BBB. In addition, few drug carriers are proteins and its deficiency (P-glycoprotein) at the BBB inhibits the efflux activity of certain biomolecules at the blood brain barrier [170].

More often, most of the drug delivery methods are based on structure activity relationships, drug-to-drug receptor interactions, and structure transport relationships mainly membrane permeation. In addition, drugs which are selected based on throughput screening may show higher binding* in silico*, but in reality* in vivo* systems invariably show poor membrane permeation because of low binding by target receptor. Therefore, it seems very difficult to pass on drug more simply through the blood barrier in theoretically relevant concentration and underlying experimental aspect of drug targeting [27]. More often, drugs undergo insignificant transport through the brain capillary endothelium that makes up the blood brain barrier (BBB)* in vitro*. But,* in vivo* system drug must bind to some specific receptor; if binding occurs partially, it will affect drug delivery. Further,* in vivo* condition absorption of drug in neuronal and other brain cells seems to be difficult after drug delivery, because its route is obstructed in many ways. Second, drug may convert into noninteracting metabolite or a portion of it might show higher binding to other ligand/s mainly proteins. Though, therapeutically, drug seems to be more appropriate by biological system but it becomes ineffective or attains some highly reactive active molecular when it reaches inside brain cells. If drug becomes able to pass through the barrier, it might adhere to the unwanted protein [171]. Another problem is created by presence of some catabolic enzymes in the brain tissues, which could change the native or active form of the drug or cleave it into an inactive molecule or slow acting pharmaceutical that may be deconstructed once it is inside the brain tissue rendering it useless. Therefore, accumulated therapeutic biomaterials in the brain play a crucial role in the pathogenesis of neuronal diseases [170]. Hence, an active penetration of drug through blood brain barrier is highly needful for its delivery at the target area in the brain for treatment of CNS disorders and diseases as well.

BBB is nonselective to pass drugs by diffusion or by active transport and is major hurdle for successful CNS drug development. But, it is true that molecules like glucose and fat/lipid soluble drugs can rapidly cross into the brain. Contrary to this, delivery of many of the drug types is very difficult to carry them into the brain because of fat insoluble. Therefore, such drugs cannot be made available to the brain because of nondelivery across the blood brain barrier. There are so many factors, which influence the drug delivery or its ability to traverse the blood brain barrier. It is true that the membrane barrier disallows larger molecules, while smaller molecules are carrying over to the brain. Similarly, charged molecules rapidly get into the brain. Therefore, lipophilicity does not seem to be necessary or the only factor that may assist the drug for safe passage to brain. There seems to be a role of multiple factors or complex molecular properties that make drug able to pass through the BBB. More exceptionally, barrier permeability is also related to membrane or luminal surface of brain capillary, composition of CSF or ISF, functional groups, change on molecular and ionic surfaces, or presence of charged residues of the molecules. In addition, surface activity of the molecules, their relative size and specific binding of transporter proteins, energy driven cassettes, and opening and closing of channels due to ionic concentration are noted as important factors [172].

Further, it is true that all traditional drug delivery methods are based on trial-and-errors. These are applied invariably for few selected drugs that had appropriate structure-activity relationships or drug-receptor interactions, and structure-transport relationships are intact. It allows drugs for suitable and successful membrane permeation. Moreover, all modern methods concerned with drug development are based on rational drug design and almost all new drugs require use of receptor-based high throughput screening methods to find appropriateness of the drug among thousands of new compounds. But, it is not true that drugs that are selected with high throughput screening solely on the basis of structure-activity relationships may show target receptor binding in animal system because they behave differently. Drug might show invariably poor membrane permeation properties* in vivo*. Such drugs will undergo insignificant transport through the brain capillary endothelium, which makes up the blood brain barrier (BBB)* in vivo*. However, several approaches for direct drug delivery or direct convection enhanced delivery are used to inject the drug into brain or cerebrospinal fluid or intranasal delivery. These techniques are highly unsafe, local, invasive, metabolizable, or short lasting. Contrary to this, drugs that are delivered through vascular route through BBB in physiologically required amount first infused and spread in larger portion of the brain. There are three different types of strategies available for drug delivery. These are neurological/direct injections, drug delivery methods. More specifically, neurosurgical, pharmacological, and physiological strategies include BBB disruption by osmotic imbalance or by using vasoactive compounds, intraventricular drug infusion, and intracerebral implants. In pharmacological methods, lipid carrier or liposomes are used for drug delivery. Physiological strategies are followed by applying endogenous transport mechanisms by using either carrier-mediated transport of nutrients or receptor-mediated transport of peptides. From clinical investigations, physiological strategies are proved better and potential delivery methods, because of wider safety cover provided by drug transport.

### 3.13. Cancer and Tumor Therapy

Similar to blood brain barrier, blood tumor barrier also imposes so many obstacles to drug delivery in the CNS. More often, brain tumor microvessels/capillaries limit drug delivery to tumors by forming a blood brain barrier [173]. Even though the blood brain tumor barrier is more permeable than the blood brain barrier [173, 174], it significantly restricts the delivery of anticancer drugs and limits systematic chemotherapeutics of brain tumors. This causes failure of drug target and made the delivery process extremely difficult to treat solid tumors in the brain. This is the main casue of clinical failures of many potential antitumor drugs, that is usually not due to lack of potency but rather it occurs due to nondelivery of drug to the brain and into the tumors [175]. In addition, pharmaceuticals used in tumor specific therapies are found insufficient to check aberrant signaling pathways in brain tumors [176]. It makes the chemotherapeutic treatment of brain tumors ineffective and required amount of drug could not be delivered into the brain [177]. Further, due to very high toxicity of antitumor chemotherapeutic drugs, these cannot be administered in sufficient concentration by conventional delivery methods. Therefore, such methods are not much helpful to ascertain long-term survival of the patients with brain tumors and most of clinical cases of brain tumors are proving fatal [177]. However, new well-designed therapeutic strategies that could make an easy and successful drug delivery should be developed to save the life of patients. Drug delivery should be more responsive for delivering an appropriate therapeutic concentration of antitumor drug by applying safer drug delivery systems or methods by beaching any physical and physiological obstacles [178].

Therefore, potential techniques are to be developed for treatment of brain tumors [175]. There are common approaches which have been followed in the past by clinicians, that is, high dose intravenous chemotherapy, intra-arterial drug delivery, local drug delivery via implanted polymers or catheters, and disruption of BBB and biochemical modulation of drug [178]. Few other drug delivery methods are intracerebro ventricular, convection enhanced delivery BBB/BTB disruption, and BTB permeability modulation [179]. Further, to enhance the BTB permeability, accelerated therapeutic molecules are allowed to pass through it by cellular vasomodulator-mediated transportation mechanism. BTB targeting specific proteins are used to increase antineoplastic drug delivery to brain tumors [179]. In addition, K (Ca) channels are potential target for biochemical modulation of BTB permeability to increase antineoplastic drug delivery selectively to brain tumors [173]. Further, BTB permeability is also enhanced with accelerated formation of pinocytic vesicles which could transport drugs across the BTB with the help of some channel activators [174]. For example, infused minoxidil sulphate (MS) is a selective K (ATP) channel activator that comes across the BTB, reaches the brain tumor and facilitates delivery of certain macromolecules mainly Her-2 antibody adenoviral-green florescent protein and carboplatin to brain tumors [173]. This antibody significantly increases the survival in rats having brain tumors. Hence, rat brain tumor models are designed to enhance drug delivery to brain tumor following intracarotid infusion of bradykinin (Bk), nitric oxide (NO) donors or agonists of soluble guanylate cyclase (SGC), and calcium dependent potassium K (Ca) channels [174]. Further, modulation of these channels by specific agonists and agents that produce NO and cGMP* in situ* is essentially required. Contrary to this, water-soluble compounds are limited by the surface area/permeability of the tumor capillaries [180]. Therefore, in new methods, BBB manipulations are being performed for safe delivery of drug to the brain, but these should be noninvasive, physiological, and pharmacological. No doubt, drug delivery into solid tumors can assist in development of novel targeted molecular based therapies for treatment of patients. It could also be possible by selective opening of blood tumor barrier by a nitric oxide donor and infusion required amount of drug into it. Certainly, safe drug delivery will increase survival and life expectancy in tumor patients [50, 181].

## 4. Conclusion

The blood brain barrier provides a layer of protection for the brain from harmful or foreign substances that may injure the brain. It also protects the brain from action of metals, toxicants, poisons, hormones, and neurotransmitters. The blood brain barrier (BBB) and blood cerebrospinal fluid barrier (BCSF) possess a variety of carrier-mediated transport systems to support and protect brain functions. These transport mechanisms are used for management of level of various substances including xenobiotic compounds by the brain. Substances with high lipid solubility may move across the blood brain barrier by simple diffusion but non lipid soluble substances are prohibited from diffusion. BBB is a very tightly packed endothelial construction that disallows large molecules to pass through it but allows smaller molecules to pass through tight junctions. More often, glucose and other low molecular fat-soluble molecules are also disallowed but few lipid soluble molecules such as barbiturate drugs rapidly cross the BBB and reach the brain. More specifically, movement of molecules with high electrical charge is slowed down. Morphologically, the polar distribution of transport proteins mediates amino acid homeostasis in the brain. Furthermore, endothelial cells that line cerebral microvessels also maintain microenvironment for reliable neuronal signaling and regulate transport of essential molecules. Similarly, BBB exerts the greatest control over the immediate microenvironment of brain cells.

No doubt, BBB like other barriers protects organs from harmful substances (e.g., xenobiotics) including drugs and foreign compounds and from xenobiotics and noxious agents. It regulates the movement of ions, molecules, and cells between the blood and CNS. It plays a crucial role in maintaining the appropriate environment for proper neural function as well as protecting the brain from injury and disease. BBB separates the pools of neurotransmitters and neuroactive agents that act centrally and peripherally and regulates the ionic microenvironment present inside neurons. BBB assists in healthy functioning of the brain and makes possible, easy and uninterrupted supply of various molecules and ions. These molecules and ions maintain life support system and physiologically protect the neurocompartments from potential disruptive and damaging effects of xenobiotic agents. Moreover, BBB protects the brain from toxic substances and restricts its entry into the neurocompartment. Thus, most of the neuroprotective drugs should improve the brain function only by toning up BBB and CNS rather than targeting neurovascular units. These drugs may open the BBB's tight junctions and allow the passage of growth factors and antibodies into the brain and reduce inflammation which causes oedema. Therefore, for therapeutic purposes and protection of CNS, integrity of blood brain barrier is highly important for having better clinical outcomes in case of neural diseases.

Further, molecular events and protein complexes involved in release of neurotransmitters by exocytosis should be known because involvement of neurotransmitters may also induce modulatory functions in many neuronal circuits. Furthermore, physiological stress response to gases such as CO_2_ and clustered nursing intervention can minimize chances of strokes and brain ischemia. More specifically, neuronal cells require both electrolytes and nonelectrolytes for performing nerve transmission but electrolytes play a vital role in the maintenance of osmotic pressure and acid base equilibrium in matrix. Mainly, Mg^2+^ ions and phosphates maintain this property. Contrary to this, nonelectrolytes normally occuring in ionizing state are Na, K, Mg, Cu, I, Fe, Mn, Fl, Mo, Cl, Zn, Co, Ni, and so forth. These perform various cellular functions and act as important cofactors for certain catabolic enzymes or associating apoenzymatic parts of many vitamins. Metal ions are also component part of some hormones, lipids, proteins, nucleic acids, sugar phosphates, and nucleoside phosphates. The iron as an important metal ion occurs in the hemoglobin, ferritin, cytochromes, and some enzymes as catalase and cytochrome oxidase, while calcium ions occur in the blood, matrix and bone. The copper (Cu), manganese (Mn), molybdenum (Mo), and zinc (Zn) are useful as cofactors for enzyme actions, while iodine and fluorine are essential for the thyroid and the enamel metabolism. Both cell cytoplasm and matrix are an enlarged depository of various kinds of ions. These are highly essential for maintaining osmotic pressure and acid base balance in the cells. However, retention of ions in the matrix produces an increase in osmotic pressure and allows the entrance of water in the cell.

Further, monocarboxylate transporters (MCTs) play a major role in the maintenance of the glycolytic metabolism through the proton-linked transmembrane transport of lactate [60]. In addition, carrier-mediated transfer systems, that is, ATP powered pumps (ATP) cassettes channels and molecular drug transporters, are used to support transport of drugs and nourishments to protect and maintain vital brain function. Moreover, role of P-glycoproteins or transmembranous proteins, ATP-dependent pumps, transporters, and permeablitizers in transportation of various nutrients and other essential elements is well defined. Hence, ATP-dependent efflux drug transporter such as Bcrp (breast cancer resistance protein) with diverse spectrum of hydrophilic and hydrophobic substrates ranging from anticancer, antiviral, and antihypertensive drugs should be used. Further, therapeutic impact of MCT transporters and its expression in plasma membrane of glioblastomas should be confirmed because monocarboxylic acid transporters control the transport of short chain monocarboxylic and keto acids, including pyruvate and lactate to support brain energy metabolism. It also regulates vesicular trafficking of MCTs in cerebrovascular endothelial cells. Therefore, for developing novel therapeutic approaches for treating brain diseases such as stroke neuronal functions, both MCT and its inhibitors should be known. These MCT inhibitors have a role in prevention of proliferation of T lymphocytes that confirms and work as promising pharmacological targets including cancer therapy.

Further, alternative methods such as disruption or loosening of BBB may assist in diffusion of most essential nutrients and biomaterials into the brain. Similarly, delivery of drugs and other substances could be possible by different carriers and drug transporters but seems to be a challenging task in clinical research. Moreover, K (Ca) channel modulation also assists in enhanced permeability of macromolecules into the brain. These are considered as promising target for biochemical modulation of BTB permeability and increase the antineoplastic drug delivery selectively to brain tumors. Moreover, K (Ca) channel are also used to deliver Her-2 monoclonal antibody and green fluorescent protein-adenoviral vectors selectively to brain for curing neoplastic, neurogenerative, and viral diseases. Further, for an enhanced drug delivery to the brain, tumor intracarotid infusion of bradykinin (BK), nitric oxide (NO) donors, or agonists of soluble guanylate cyclase (sGC) and calcium-dependent potassium (K (Ca)) channels were found more effective and safer. Interestingly, nitric oxide (NO) is implicated in the regulation of vascular permeability and blood flow. Bcrp is an integral protein in cancer and normal cells are a major component of the BBB located on the endothelial cells near tight junctions. Similarly, multidrug resistant protein 4 shows low permeability across the BBB resulting in low distribution to the brain. Therefore, brain to blood efflux transport systems also play important role in the cerebral clearance of endogenous neurotoxic compounds such as prostaglandins and beta amyloid that can reduce the physiological effects of CNS related disorders. Further, by management of endogenous and xenobiotic compounds by the brain permeability, functions of BBB are to be reexplored. Further, regulation of oxygen metabolism, plasminogen activator, thrombolytic drugs after stroke, peroxisome-proliferator-activated receptors, and inhibitors of kinase pathways could also assist in drug delivery. In addition, after use of drugs or other metabolites or CNS, formed neurotoxicants vesicles play important role in its release. Interestingly, vesicles protect cells from accumulation of wastes or drugs and show many clinical applications ranging from biomarkers to anticancer therapy. Therefore, alternate methods of drugs delivery and substance transport should be practiced to find an appropriate solution of CNS related pathogenicity and neurodegenerating diseases. Hence, to fulfill drug delivery tasks, better understanding is required among clinicians and immunologists for starting new research initiatives to make landmark innovations in the field of pharmacology, drug delivery, and clinical therapeutics of CNS related diseases

In addition, molecular interactions with biological barriers should be properly investigated in experimental animals as well as* in vitro* systems to develop highly efficient drug delivery systems. Moreover, advanced methods are essentially required for easy delivery of healers, peptides, proteins, growth factors, vaccines, and antibodies for neuroprotection against CNS diseases. Therefore, collaborative research efforts should be made by clinicians and molecular biologists to develop/explore new innovative methods of drug delivery. Further, there seems an instant need of new smaller pharmaceuticals having target specific designs to protect the CNS from various pathological impairments. Hence, use of natural transporters such as ABC cassettes, aquaporins, cationic pumps, and substance specific ionic channels should favor to support transport of drugs and nourishments to maintain vital brain functions. Moreover, drugs that can enhance permeability functions of nerve cell plasma membrane should be promoted. It will not only help to deliver the pharmaceuticals but also assist in finding new signaling pathways for transport of substances mainly fuel materials and drugs for successful treatment of infectious viral diseases. Further, normal supply of substances could maintain integration of CNS functions such as learning, memory, sleep cycle, movement, hormone regulation, vision, hearing, and many other functions. More often, after exploring transcellular signaling pathways generated by beneficial drug candidates, its action and authenticating behavior could be assessed much easily. Hence, strong recommendations are being made to upgrade pharmaceutical technologies by combining them with the recent advances in membrane biology and clinical sciences to extend the therapeutic use of pharmaceuticals for treatment of neurological diseases and CNS impairments. Further, biomedical researchers should increase the spectrum of pharmaceuticals by carrying them to targeted locations. Thus, by developing new endothelial transporters/carriers and new drug candidates, a rapid solution of neurodegenerative and neuropathological disease and CNS protection could be achieved. Further,* in vitro* high throughput screens and computational BBB tools together with understanding of brain penetration may enable the drug discovery community to minimize the access of drug candidate into the CNS compartment. Therefore, compounds which can reduce apoptosis, oxidative stress, induce neuritis, outgrowth, synaptic plasticity, and memory loss due to detrimental effects imposed by xenobiotics and pathogens should be of greater use. No doubt, new upgraded drug delivery systems and therapeutic candidates will provide novel treatment for different types of CNS and neurodegenerative diseases [182].

## Figures and Tables

**Table 1:** Cellular functions of various kinds of metal and nonmetal ions inside cell and its transport mechanisms.

Element	Ionic form	Transport mechanism	Functions	References

Molybdenum	MoO_4_^2^	Ion transporters	Cofactor or activator of certain enzymes (e.g., nitrogen fixation, nucleic acid metabolism, and aldehyde oxidation)	

Cobalt	Co^2+^	Ion transporters, DMT1	Constituent of vitamins B_12_	

Copper	Cu^+^, Cu^2+^	ATP 7A catalyzes, Cu-transporting ATPase	Constituent of plastocyanin and cofactor of respiratory enzymes.	[28–32]

Iodine (heaviest trace element)	I	Diffusion and ion transporters	Constituent of thyroxin, triiodothyronine, and other thyroid hormones.	

Boron	BO_3_^3−^, B_4_O^−2^	Ion transporters	Activates arabinose isomerase.	

Zinc	Zn^2+^	Enzyme and protein carriers	Cofactor of certain enzymes (e.g., carbonic anhydrase, carboxypeptidase)	

Manganese	Mn^2+^, (Mn^2+^).[4]	DMT1	Cofactor of certain enzymes (e.g., several kinases, isocitric decarboxylase).	

Iron	Fe^2+^, Fe^3+^	DMT1	Constituent of haemoglobin, myoglobin, and cytochromes.	

Magnesium	Mg^2+^	Ion transporters	Constituent of chlorophyll activates ATPase enzyme	

Sulphur	SO_4_^2^	Ion transporters	Constituent of coenzyme A, biotin, thiamine, and proteins.	

Phosphorus	PO_4_^3−^, H_2_PO_4_	Ion transporters	Constituent of lipids, proteins, nucleic acids, sugar phosphates, and nucleoside phosphates.	

Calcium	Ca^2+^	Ion transporters	Constituent of plant cell walls; matrix component of bone tissue; cofactor of coagulation enzymes.	

Potassium	K^+^	Diffusion and ion channels	Cofactor for pyruvate kinase and K^+^-stimulated ATPase.	

Sodium	Na^+^	Diffusion and ion channels	Perform nerve functions, acid base balance, and homeostasis	

Cadmium	Cd^2+^	DMT1		

Zinc^2+^	Zn, ZIP6 (LIV-1)	Zinc transporters	Metabolic cellular functions	[33]

Aluminium	Al	Transferrin-receptor mediated endocytosis		

Metal ions	Fe, Cu, and Ag	Multiple transport proteins, Na^+^/K^+^ pump, K^+^ channel, Na^+^ lysine symporter channel, and sodium-lysine transporter	Work as electrolyte in plasma	[34]

Metal ions	Fe, Cu, and Ag	Membrane transporter proteins such as ABC transport proteins	Enzymatic cellular functions	[35]

Free metal ion and complexes	Fe, Cu, Ag oxides, and sulphates	Transferrin carries metal species, such as the free metal ion and complexes of the metal with an amino acid or protein.	Transmigration of proteins	[36, 37]

Divalent metals	Fe, Cu, Ag, and zinc	(DMT1, DCT1, and Nramp2) transports, ferroprotein,		[36, 38, 39]

Divalent metals	Fe3+ and cobalt (Co2+), Ca++, Fe++, Mn++, Cd++, Cu++, Ni++, Pb++, Zn ++	Divalent metal transporter 1, divalent cation transporter 1 (DCT1)	Iron-overload disorders	[40, 41]

Trivalent metals	Al and Mn	Diferric transferrin		[42]

Methyl mercury		Transport by the L-cysteine system		[43]

**Table 2:** Transport mechanisms of gases, solvents, and substances across blood brain barrier.

Type of substance/gas	Transporter	Location	Functions	References

Small inorganic molecules, that is O_2_, CO_2_, NO, and H_2_O	Diffusion	Ionic pores/receptors	Influx or efflux	[47]

Oxygen	Diffusion	Ionic pores/receptors	Vitality, homeostasis, and cellular metabolism	[48]

Ammonia	Passive diffusion	Ionic pores/receptors	Irritation, toxic	[49]

Nitric oxide	Passive diffusion	Ionic pores/receptors	Toxic	[50]

Water	Diffusion and by aquaporins 1–5	Membrane surface aquaporins	Controls water relations, regulators of transcellular water flow	[51–54]

Ethanol	By filtration moving through water spaces	Ionic pores	Alcohol-induced oxidative/nitrosative stress alters brain mitochondrial membrane properties and alters basal extracellular glutamate concentrations and clearance in the mesolimbic system	[55–57]

Solutes	Paracellular or transcellular diffusion	Luminal surface	Influx or efflux	[58, 59]

Glucose, hexose (GLUT-1)	Facilitated diffusion by GLUT 1 and GLUT 3 Transporters	Glucose, morphine-6-*β*-D glucuronide	Influx and efflux monosaccharide transport Proteins play important role in carbohydrate assimilation, distribution, metabolism, and homeostasis	[44–46]

Monocarboxylate	Proton linked transport	Maintenance of glycolytic metabolism	Proliferation of T-lymphocytes	[60, 61]

Monocarboxylate (MCTI)	Facilitated diffusion	Benzoic, lactic, lovastatin, and pyruvic acid	Influx and efflux promising pharmacological targets including for cancer chemotherapy	[61, 62]

Anion exchange (band 3)	Facilitated diffusion	Cl^−^/HCO_3_^−^, lactate, and metal-anion com-	Influx and efflux	

Tf-FMMs transferring-conjugated fluorescein loaded magnetic nanoparticles	Receptor mediated transcytosis	Nanoparticles	Influx, drug delivery	[63]

Polycationic proteins and lectins	Absorptive-mediated transcytosis	Membrane receptors	Influx, therapeutic	[64]

Peptide	Saturable or facilitated transport and *transmembrane diffusion* or passive diffusion	Membrane receptors	Influx, therapeutic	[64]

Saturated fatty acids	Facilitated diffusion	Octanoate	Influx	

Urea	Facilitated diffusion		Efflux	

Xenobiotics	Xenobiotics transporters	Ionic pores/receptors	Out flux, toxicity	[65–67]

Xenobiotics. 1-methyl-4-phenylpyridinium (MPP+)	Uptake2 (Oct1-3/Slc22a1-3, Pmat/Slc29a4), Net, and Mate1/Slc47a1 transporters	ATP-driven xenobiotic efflux transporters		[35, 68, 69]

**Table 3:** Vitamins and their transport across the blood brain barrier (BBB); mechanisms and functions.

Fat-soluble	Transport mechanism	Physiological functions	Deficiency disease	References

Vitamin A (retinol and retinoic acid)	Vitamins show specific high-affinity to proteins which transport them. These are also transported after being mixed with lipid micelles formed with the aid of bile salts, lysolecithin, lower glycerides, and cholesterol.	Stored in liver; maintains general health and vigor of epithelial cells.	Xerophthalmia	[59, 78]

Vitamin D (calciferol)	Vitamin D and its hydroxylated metabolites are transported in the blood, bound to a transport protein DBP which is also very important in the placental transfer of 25-hydroxy-vitamin Dl.	Involved in intestinal absorption of calcium and phosphorus and in calcium metabolism and bone formation.	Rickets and osteomalacia; it also affects brain development, function and its low levels cause neuropsychiatric diseases like autistic spectrum disorder and schizophrenia. Its deficiency in early life affects neuronal differentiation, axonal connectivity, dopamine ontogeny, and brain structure and function.	[76, 79]

Vitamin E (Tocopherol)	Intestinal absorption and transport occur with chylomicrons. Alpha-tocopherol is mostly transferred to parenchymal cells of the liver where it is stored.	Inhibit catabolism (i.e., oxidation) of certain fatty acids of cellular membranes.	Hemolytic anemia affects brain functions mainly related to mitochondrial and peroxisomes.	[80]

Vitamin K (naphthoquinone)	Vitamin K is a quinone compound in the human body in a storage form as menaquinone (MK); distribution includes regulated amounts in mitochondrial membranes. The human brain, which has low amounts of typical vitamin K dependent function (e.g., gamma carboxylase) has relatively high levels of MK, and different regions of brain have different amounts.	Required for the formation of prothrombin (an essential component of blood clotting). Coenzyme Q is a quinone synthesized *de novo*, and the levels of synthesis decline with age. The levels of MK are dependent on dietary intake and generally increase with age.	Sever bleeding during delivery, delayed blood clotting, MK has a characterized role in the transfer of electrons to fumarate in prokaryotes. A newly recognized fumarate cycle has been identified in brain astrocytes.	[81]

**Water Soluble Vitamins** Vitamin B (thiamine)	By diffusion	Essential for synthesis of acetylcholine; rapidly destroyed by heat, carbohydrate metabolism.	Beriberi, polyneuritis affect impulse transmission, thoughts, and intelligence.	

Vitamin B_2_ (riboflavin)	By diffusion	Forms the coenzyme FAD which is involved in metabolism of carbohydrates and proteins.	Dermatitis, blurred vision.	

Niacin (nicotinic acid)	By diffusion	Forms the coenzyme NAD which is involved in energy-releasing reactions. In lipid metabolism, it inhibits production of cholesterol and helps in fat break down.	Pellagra	

Vitamin **B**6 (pyridoxine)	Transported across brain and choroid plexus in nonphosphorylated B form and has separate carriers. The vitamin B6 derivative pyridoxal phosphate is a cofactor in the synthesis of GABA.	Forms coenzymes involved in amino acid metabolism in brain and is involved in fat metabolism; it imposes inhibitory effects on the brain neurons that show protective role during hypoxia or ischemia.	Convulsion, both cofactor and noncofactor vitamin (e.g., of B(1), B(3), B(6), and E) have potential role in the therapy of brain disorders.	[77]

Folic acid	By diffusion, and protein transporters	Essential for synthesis of DNA; overcooking destroys it.	Macrocytic anaemia	

Biotin (vitamin H)	By diffusion and protein transporters	Acts as coenzyme in metabolism of carbohydrates, fatty acids, and nucleic acid.	Mental depression, muscular pain, dermatitis, and nausea	

Vitamin B_12_ (cyanocobalamin)	Vitamin B12 permease ABC protein transporter; it transports protein containing two transmembrane domains (T) and two cytosolic ATP binding domain.	Acts as coenzyme necessary for DNA synthesis, red blood cell for motion, growth, and nerve function.	Malfunctioning of CNS	

Vitamin C (ascorbic acid)	Absorbs through diffusion by glucose GLUT1 transporter, glucose GLUT1 also transports dehydroascorbic acid into the brain and its transport is inhibited by d-glucose; Slc23a1 is ascorbic-acid transporter that is a Na^+^-dependent system.	Promotes protein synthesis (collagen), wound healing, and iron absorption; protects body against infections; rapidly destroyed by heat.	Scurvy: vitamin C crosses the placenta and is an essential requirement in the perinatal period.	[82, 83]

**Table 4:** Major neurotransmitter transporters occur at blood brain barrier.

Neurotransmitters	Transporter/mechanism	Location	Influx or efflux/function	References
**Neurotransmitters**	Diffusion	Membrane of presynaptic and postsynaptic neuron fiber	Influx	[86]

**Choline**	Carrier-mediated process	Membrane of presynaptic and postsynaptic neuron fiber	Influx and efflux regulate the formation of acetylcholine in the central nervous system.	[87]

**Acetylcholine**	Facilitated diffusion, saturable glycoproteins (Pgp) transport system	Neuronal circuits of brain, membrane of presynaptic, and postsynaptic neuron fiber	Influx, Synaptic transmission, occurs in peripheral nervous system.	[86, 88–90]

**Glycine**	Receptor mediated	Membrane receptors	Makes the postsynaptic membrane more permeable to Cl-ions.	

**Aspartate**	Aspartate ion channel	Membrane receptors		

**Glutamate**	Glutamate chloride-independent membrane transport system	Membrane receptors		

**Glutamate/asparate**	ATP-driven Na^+^-K^+^-ase	Membrane receptors		

**Biogenic amines, norepinephrine**	**Norepinephrine transporter**, uptake1 and uptake2 carrier-mediated transporters, Net (Slc6a2), Dat (Slc6a3), and Sert (Slc6a4)	Norepinephrine	Norepinephrine transporter Alzheimer's disease and attention deficient hyperactivity disorder	[68, 92, 93] [94–96]; [97]

**Melatonin**		Membrane receptors	Melatonin receptors in the area postrema located outside the blood-brain barrier	[98, 99]

**Peptides**		Membrane receptors		[100]

**Cholecystokinin-1 receptor agonist**	Cholecystokinin-1 receptor agonist	Membrane receptors		[101, 102]

**GI hormone **		Membrane receptors		[103, 104]

**Insulin, transferrin, insulin-like growth factors and vasopressin**	Receptor-mediated transcytosis	Membrane receptors		[105]

Serotonin		Serotonin		

GABA (GAT2, BGT1)	Amino acid transporter	GABA	GABA imposes inhibitory effects on the brain neurons that show protective role during hypoxia or ischemia, active transport into the astrocyte glial cells	

**Nucleoside transporters**				

Equilibrative nucleoside transporters (ENT*es* & ENT*ei*)	Nucleoside transporters	Nucleoside	Purine and pyrimidine nucleosides, purine, and pyrimidine nucleosides	

**Ion transporters**				

Na/K-ATPase	Active transport	Potassium	Nerve conduction	

Ca-ATPase	Active transport	Calcium	Bone marrow	

Sodium	Active transport	Sodium	Nerve conduction	

Potassium, chloride	Active transport	Potassium/chloride cotransporter	Nerve conduction	

**ATP-binding cassette (ABC) transporters**				

P-glycoprotein (P-gp) multidrug resistance transporter (mouse Mdr1a [Mdr3], Mdr1a [Mdr3], Mdr1b [Mdr1], and Mdr2; human MDR1 & 2)	Active transport	Organic, hydrophobic, and amphipathic cations: *β*-adrenergic blockers, anthracyclines, cyclosporine A, digoxin, etoposide, glucocorticoids, loperamide, morphine, phenytoin, vecuronium, verapamil, and vinca alkaloids	Inhibition of drug transport	

Multidrug resistance associated protein (MRP1)	Active transport	Amphipathic anions and glutathione, glucuronide and sulfate conjugates: benzylpenicillin, doxorubicin, etoposide, methotrexate, vinblastine, and vincristine	Efflux	

MRP2 (c-MOAT)	Active transport	Organic anions	Efflux	

MRP3 (MOAT-D)	Active transport	Methotrexate, anionic conjugates	Efflux	

MRP4 (MOATB)	Active transport	Nucleoside-based antivirals	Efflux	

MRP5 (MOAT-C)	Active transport	Cyclic nucleotides, glutathione conjugates, and organic anions	Efflux	

MRP6 (MOAT-E)	Active transport	Peptides	Efflux	

Breast cancer resistance protein BCRP)	Active transport	Similar to P-gp substrates; camptothecin derivatives, daunorubicin, doxorubicin, mitoxantrone, and topotecan	Efflux	

OCT1-3,	Active transport	Monoamine neurotransmitters, cationic neurotoxins, amantadine, memantine, desipramine, dopamine, and serotonin	Influx and efflux	

OCTN1	Active transport	Antiarrhythmic drugs, tetraethylammonium verapamil	Influx and efflux	

OCTN2		*β*-lactam antibiotics, L-carnitine, and tetraethylammonium	Influx and efflux	

Organic ion transporting polypeptide 1 (oatp1, OATP1)	Active transport	Bulky organic cations, glucuronide conjugates, steroid hormones, estrone sulfate, indinavir, nelfinavir, opioid agonists, and ouabain	Influx and efflux	

Oatp2, OATP2	Active transport	Amphipathic anions: bile acids, cholate, digoxin, estrogen conjugates, opioid agonists, and ouabain, taurocholate; dehydroepiandrosterone sulfate and estrone-1-sulfate efflux; [26]-enkephalin; 3,5,3-triiodo-L-thyronine	Influx and efflux	

Oatp3	Active transport	Digoxin, [26]-enkephalin, fexofenadine 3,5,3-triiodo-L-thyronine	Influx and efflux	

BBB-specific anion transporter type 1 (Bsat1, oatp14)	Active transport	T4	Influx and efflux	

OATP-A, OAT1, OAT3	Active transport	Amphipathic anions, bile acids, deltorphin II, estrone-1-sulfate, fexofenadine, opioid peptides, and benzylpenicillin Cimetidine, dehydroepiandrosterone sulfate, estrone sulfate, indoxyl sulfate, and PAH	Influx and efflux	

**Table 5:** Major amino acid transporters occur at blood brain barrier.

Type of amino acid	Transporter	Location	Influx or efflux	References

Influx	Amino acid carrier system or alanine preferring transporter and 1-dihydroxy-phenylalanine (1-DOPA).	Antiluminal surface of the brain capillary		[123, 124]

Alanine, glycine, proline, and *γ*-aminobutyric acid	A-(alanine-preferring-) system carrier	Antiluminal surface of the brain capillary	Influx	[64, 125]

Small neutral amino acids, (system A), L-alanine, L-asparaginine, L-cysteine, glutamine, glycine, L-histidine, L-proline, and L-serine	A-system carrier, secondary active transport	Antiluminal surface of the brain capillary	Influx, efflux	[126]

Basic, acidic and *β*-amino acids; glutamate, aspartate, and *β*-amino acid taurine	Simple carrier system	Membrane receptors	Influx	[127, 128]

L-proline	Na^+^-gradient-dependent transport	Membrane receptors		[129]

Large neutral amino acid (system L) (LAT1 and LAT2)	Facilitated diffusion	Branched or aromatic side chain aminoacids; L-DOPA, *α*-methyl-DOPA, gabapentin, L-leucine, melphalan, L-phenylalanine, L-tryptophan, and L-tyrosine	Influx	

Na^+^-LNAA neutral amino Acid	A-system carrier, secondary active transport	Alanine, glycine, histidine, isoleucine, leucine, methionine, phenylalanine, threonine, tryptophan, tyrosine, and valine	Efflux	

Neutral amino acid system ASC) (ASCT1 and ASCT2)	A-system carrier, secondary active transport	L-alanine, L-aspartate, L-cysteine, L-glutamate, glycine, L-isoleucine, L-leucine, L-methionine, L-serine, L-threonine, and L-valine	Efflux	

System B0+	Simple carrier system	Basic and neutral amino acids	Efflux	

System *β*	Simple carrier system	*β*-alanine, taurine		

Basic amino acid	Simple carrier system	Lysine	Influx	

Acidic amino acid (system x−)	Facilitated diffusion	L-aspartate, L-glutamate		

System X−	Simple carrier system	L-aspartate, L-glutamate	Efflux	

N-system	A-system carrier, Secondary active transport	L-asparaginine, glutamine, glutamate, L-histidine, and L-serine		

Excitatory amino acid (EAAT1-3)	Simple carrier system	Aspartate, glutamate	Influx	

Amine (cation, system y+)	Active transport	L-arginine, choline, L-cysteine, lysine, and ornithine	Influx	

System T	Simple carrier system	Thyroid hormones T3 and T4		

## Abbreviations

BBB: Blood brain barrier
CNS: Central nervous system
CSF: Cerebrospinal fluid
GLUT: Glucose transporter
GABA: Gama amino butyric acid
MSG: Monosodium glutamate
NVU: Neurovascular unit
DMT: Divalent metal transporter
CURL: Uncoupling receptor and ligand
RME: Receptor-mediated endocytosis
Bk: Bradykinin
NO: Nitric oxide donors
SGC: Soluble guanylate cyclase
MCT: Monocarboxylate transporters
DOPA: Dihydroxy-phenylalanine
AME: Absorptive-mediated
Tf-FMMs: Transferring-conjugated fluorescein loaded magnetic nanoparticles.

## References

[1] Bradbury M. W. B. (1997). Transport of iron in the blood-brain-cerebrospinal fluid system. *Journal of Neurochemistry*.

[2] Barres B. A. (2003). What is a glial cell?. *GLIA*.

[3] Barres B. A. (2008). The mystery and magic of glia: a perspective on their roles in health and disease. *Neuron*.

[4] De Keyser J., Mostert J. P., Koch M. W. (2008). Dysfunctional astrocytes as key players in the pathogenesis of central nervous system disorders. *Journal of the Neurological Sciences*.

[5] Seifert G., Schilling K., Steinhäuser C. (2006). Astrocyte dysfunction in neurological disorders: a molecular perspective. *Nature Reviews Neuroscience*.

[6] Sofroniew M. V. (2000). Astrocyte failure as a cause of CNS dysfunction. *Molecular Psychiatry*.

[7] Sofroniew M. V. (2005). Reactive astrocytes in neural repair and protection. *Neuroscientist*.

[8] Sofroniew M. V. (2009). Molecular dissection of reactive astrogliosis and glial scar formation. *Trends in Neurosciences*.

[9] Takano T., Oberheim N. A., Cotrina M. L., Nedergaard M. (2009). Astrocytes and ischemic injury. *Stroke*.

[10] Beck D. W., Vinters H. V., Hart M. N., Cancilla P. A. (1984). Glial cells influence polarity of the blood-brain barrier. *Journal of Neuropathology and Experimental Neurology*.

[11] Mok K. M., Lie P. P., Mruk D. D. (2012). The apical ectoplasmic specialization-blood-testis barrier functional axis is a novel target for male contraception. *Advances in Experimental Medicine and Biology*.

[12] Kanwar J. R., Sriramoju B., Kanwar R. K. (2012). Neurological disorders and therapeutics targeted to surmount the blood-brain barrier. *International Journal of Nanomedicine*.

[13] Jain A., Jain A., Gulbake A., Shilpi S., Hurkat P., Jain S. K. (2013). Peptide and protein delivery using new drug delivery systems. *Critical Reviews in Therapeutic Drug Carrier Systems*.

[14] Daneman R. (2012). The blood-brain barrier in health and disease. *Annals of Neurology*.

[15] Bérézowski V., Mysiorek C., Kuntz M., Pétrault O., Cecchelli R. (2012). Dysfunction of the blood-brain barrier during ischaemia: a therapeutic concern. *Biologie Aujourd'hui*.

[16] Wager T. T., Liras J. L., Mente S., Trapa P. (2012). Strategies to minimize CNS toxicity: *in vitro* high-throughput assays and computational modeling. *Expert Opinion on Drug Metabolism and Toxicology*.

[17] Rubin L. L., Staddon J. M. (1999). The cell biology of the blood-brain barrier. *Annual Review of Neuroscience*.

[18] Fang L., Wang M., Gou S., Liu X., Zhang H., Cao F. (2014). Combination of amino acid/dipeptide with nitric oxide donating oleanolic acid derivatives as PepT1 targeting antitumor prodrugs. *Journal of Medicinal Chemistry*.

[19] Su C. K., Yang C. H., Lin C. H., Sun Y. C. (2014). In-vivo evaluation of the permeability of the blood-brain barrier to arsenicals, molybdate, and methylmercury by use of online microdialysis-packed minicolumn-inductively coupled plasma mass spectrometry. *Analytical and Bioanalytical Chemistry*.

[20] Kuittinen O., Siniluoto T., Isokangas M. (2013). Chemotherapy in conjunction with blood brain barrier disruption in the treatment of primary central nervous system lymphoma. *Duodecim*.

[21] Bae Y., Fukushima S., Harada A., Kataoka K. (2003). Design of environment-sensitive supramolecular assemblies for intracellular drug delivery: polymeric micelles that are responsive to intracellular pH change. *Angewandte Chemie*.

[22] Packhaeuser C. B., Schnieders J., Oster C. G., Kissel T. (2004). In situ forming parenteral drug delivery systems: an overview. *European Journal of Pharmaceutics and Biopharmaceutics*.

[23] Vandermeulen G. W. M., Klok H. (2004). Peptide/protein hybrid materials: enhanced control of structure and improved performance through conjugation of biological and synthetic polymers. *Macromolecular Bioscience*.

[24] Purves D., Augustine G. J., Fitzpatrick D. (2008). *Neuroscience*.

[25] Secko D. (2006). Breaking down the bloodbrain barrier. *Canadian Medical Association Journal*.

[26] Ramlakhan N., Altman J. (1990). Breaching the blood-brain barrier. *New Scientist*.

[27] Dadparvar M., Wagner S., Wien S. (2011). HI 6 human serum albumin nanoparticles-Development and transport over an *in vitro* blood-brain barrier model. *Toxicology Letters*.

[28] Yoshimura N., Kida K., Usutani S., Nishimura M. (1995). Histochemical localization of copper in various organs of brindled mice after copper therapy. *Pathology International*.

[29] Yoshimura N. (1994). Histochemical localization of copper in various organs of brindled mice. *Pathology International*.

[30] Kodama H., Abe T., Takama M., Takahashi I., Kodama M., Nishimura M. (1993). Histochemical localization of copper in the intestine and kidney of macular mice: light and electron microscopic study. *Journal of Histochemistry and Cytochemistry*.

[31] Qian M., Tu C., Earnhardt J. N., Laipis P. J., Silverman D. N. (1997). Glutamate and aspartate as proton shuttles in mutants of carbonic anhydrase. *Biochemistry*.

[32] Qian Y., Tiffany-Castiglioni E., Harris E. D. (1995). Copper transport and kinetics in cultured C6 rat glioma cells. *The American Journal of Physiology—Cell Physiology*.

[33] Colvin R. A. (1998). Characterization of a plasma membrane zinc transporter in rat brain. *Neuroscience Letters*.

[34] Redzic Z. (2011). Molecular biology of the blood-brain and the blood-cerebrospinal fluid barriers: similarities and differences. *Fluids and Barriers of the CNS*.

[35] Wang X., Sykes D. B., Miller D. S. (2010). Constitutive androstane receptor-mediated up-regulation of ATP-driven xenobiotic efflux transporters at the blood-brain barrier. *Molecular Pharmacology*.

[36] Yokel R. A. (2006). Blood-brain barrier flux of aluminum, manganese, iron and other metals suspected to contribute to metal-induced neurodegeneration. *Journal of Alzheimer's Disease*.

[37] Laterra J., Keep R., Betz L. A., Goldstein G. W. (1999). Blood—brain barrier. *Basic Neurochemistry: Molecular, Cellular and Medical Aspects*.

[38] Ansar M., Serrano D. I., Papademetriou T. K., Bhowmick S. M. (2013). Biological functionalization of drug delivery carriers to bypass size restrictions of receptor—mediated endocytosis independently from receptor targeting. *ACS Nano*.

[39] Brewer E., Lowman A. M. (2014). Assessing the transport of receptor-mediated drug-delivery devices across cellular monolayers. *Journal of Biomaterials Science: Polymer*.

[40] Howitt J., Putz U., Lackovic J. (2009). Divalent metal transporter 1 (DMT1) regulation by Ndfip1 prevents metal toxicity in human neurons. *Proceedings of the National Academy of Sciences of the United States of America*.

[41] Magyar J. P., Bartsch U., Wang Z. Q. (1994). Degeneration of neural cells in the central nervous system of mice deficient in the gene for the adhesion molecule on glia, the beta 2 subunit of murine Na,K-ATPase. *The Journal of Cell Biology*.

[42] Eid C., Hémadi M., Ha-Duong N. T., El Hage Chahine J. M. (2014). Iron uptake and transfer from ceruloplasmin to transferrin. *Biochimica et Biophysica Acta*.

[43] Kerper L. E., Ballatori N., Clarkson T. W. (1992). Methylmercury transport across the blood-brain barrier by an amino acid carrier. *The American Journal of Physiology*.

[44] Magier Z., Jarzyna R. (2013). The role of glucose transporters in human metabolic regulation. *Postepy Biochemii*.

[45] Iwabuchi S., Kawahara K. (2013). Extracellular ATP-prinoceptor signaling and AMP-activated protein kinase regulate astrocytic glucose transporter 3 in an *in vitro* ischemia. *Neurochemistry International*.

[46] Cura J. A., Carruthers A. (2012). Role of monosaccharide transport proteins in carbohydrate assimilation, distribution, metabolism, and homeostasis. *Comprehensive Physiology*.

[47] Burton P. S., Conradi R. A., Ho N. F. H., Hilgers A. R., Borchardt R. T. (1996). How structural features influence the biomembrane permeability of peptides. *Journal of Pharmaceutical Sciences*.

[48] Li J., Konstantinov I. E., Cai S., Shimizu M., Redington A. N. (2007). Systemic and myocardial oxygen transport responses to brain death in pigs. *Transplantation Proceedings*.

[49] Sørensen M. (2013). Update on cerebral uptake of blood ammonia. *Metabolic Brain Disease*.

[50] Weyerbrock A., Walbridge S., Pluta R. M., Saavedra J. E., Keefer L. K., Oldfield E. H. (2003). Selective opening of the blood-tumor barrier by a nitric oxide donor and long-term survival in rats with C6 gliomas. *Journal of Neurosurgery*.

[51] Fabrick J. A., Pei J., Hull J. J., Yool A. J. (2013). Molecular and functional characterization of multiple aquaporin water channel proteins from the western tarnished plant bug, *Lygus hesperus*. *Insect Biochemistry and Molecular Biology*.

[52] Day R. E., Kitchen P., Owen D. S. (2013). Human aquaporins: regulators of transcellular water flow. *Biochimica et Biophysica Acta*.

[53] Segal M. B., Zlokovic B. V. (1990). Transport of large peptides and proteins across the blood-brain barrier. *The Blood-Brain Barrier, Amino Acids and Peptides*.

[54] Chaumont F., Tyerman S. D. (2014). Aquaporins: highly regulated channels controlling plant water relations. *Plant Physiology*.

[55] Jiang L., Gulanski B. I., De Feyter H. M. (2013). Increased brain uptake and oxidation of acetate in heavy drinkers. *Journal of Clinical Investigation*.

[56] Ding Z., Rodd Z. A., Engleman E. A., Bailey J. A., Lahiri D. K., McBride W. J. (2013). Alcohol drinking and deprivation alter basal extracellular glutamate concentrations and clearance in the mesolimbic system of alcohol-preferring (P) rats. *Addiction Biology*.

[57] Reddy V. D., Padmavathi P., Kavitha G., Saradamma B., Varadacharyulu N. (2013). Alcohol-induced oxidative/nitrosative stress alters brain mitochondrial membrane properties. *Molecular and Cellular Biochemistry*.

[58] Karp G. (1999). *Cell and Molecular Biology, Concepts and Experiments*.

[59] Pardridge W. M., Sakiyama R., Coty W. A. (1985). Restricted transport of Vitamin D and A derivatives through the rat blood-brain barrier. *Journal of Neurochemistry*.

[60] Miranda-Gonçalves V., Honavar M., Pinheiro C. (2013). Monocarboxylate transporters (MCTs) in gliomas: expression and exploitation as therapeutic targets. *Neuro-Oncology*.

[61] Halestrap A. P. (2013). Monocarboxylic acid transport. *Comprehensive Physiology*.

[62] Moschen I., Bröer A., Galić S., Lang F., Bröer S. (2012). Significance of short chain fatty acid transport by members of the monocarboxylate transporter family (MCT). *Neurochemical Research*.

[63] Yan F., Wang Y., He S., Ku S., Gu W., Ye L. (2013). Transferrin-conjugated, fluorescein-loaded magnetic nanoparticles for targeted delivery across the blood-brain barrier. *Journal of Materials Science: Materials in Medicine*.

[64] Kotyk A., Janace K. K. (1975). *Cell Membrane Transport*.

[65] Abbott N. J., Patabendige A. A. K., Dolman D. E. M., Yusof S. R., Begley D. J. (2010). Structure and function of the blood-brain barrier. *Neurobiology of Disease*.

[66] Smith J. P., Uhernik A. L., Li L., Liu Z., Drewes L. R. (2012). Regulation of Mct1 by cAMP-dependent internalization in rat brain endothelial cells. *Brain Research*.

[67] Nicholson C. (2001). Diffusion and related transport mechanisms in brain tissue. *Reports on Progress in Physics*.

[68] Pascal A., Bruno S., Cochois-Guégan V. (2012). Transport of biogenic amine neurotransmitters at the mouse blood-retina and blood-brain barriers by uptake1 and uptake2. *Journal of Cerebral Blood Flow and Metabolism*.

[69] Koepsell H., Lips K., Volk C. (2007). Polyspecific organic cation transporters: structure, function, physiological roles, and biopharmaceutical implications. *Pharmaceutical Research*.

[70] Sharan M., Jones M. D., Koehler R. C., Traystman R. J., Popel A. S. (1989). A compartmental model for oxygen transport in brain microcirculation. *Annals of Biomedical Engineering*.

[71] Safaeian N., David T. (2013). A computational model of oxygen transport in the cerebrocapillary levels for normal and pathologic brain function. *Journal of Cerebral Blood Flow & Metabolism*.

[72] Gotoh F., Tazaki Y., Meyer J. S. (1961). Transport of gases through brain and their extravascular vasomotor action. *Experimental Neurology*.

[73] Genzler L., Johnson P. J., Ghildayal N., Pangarakis S., Sendelbach S. (2013). End-tidal carbon dioxide as a measure of stress response to clustered nursing interventions in neurologic patients. *American Journal of Critical Care*.

[74] Wang S., Shi Y., Shu S., Guyenet P. G., Bayliss D. A. (2013). Phox2b-expressing retrotrapezoid neurons are intrinsically responsive to H^+^ and CO_2_. *Journal of Neuroscience*.

[75] Sachdeva R., Singh B. (2014). Insights into structural mechanisms of gating induced regulation of aquaporins. *Progress in Biophysics and Molecular Biology*.

[76] Eyles D. W., Burne T. H. J., McGrath J. J. (2013). Vitamin D, effects on brain development, adult brain function and the links between low levels of vitamin D and neuropsychiatric disease. *Frontiers in Neuroendocrinology*.

[77] Spector R., Johanson C. E. (2007). Vitamin transport and homeostasis in mammalian brain: focus on vitamins B and E. *Journal of Neurochemistry*.

[78] Glover J., Walker R. J. (1964). Absorption and transport of vitamin A. *Experimental Eye Research*.

[79] Bouillon R., Van Baelen H. (1980). The transport of vitamin D. *Pediatric Research*.

[80] Drevon C. A. (1991). Invited review: absorption, transport and metabolism of vitamin E. *Free Radical Research Communications*.

[81] Lovern D., Marbois B. (2013). Does menaquinone participate in brain astrocyte electron transport?. *Medical Hypotheses*.

[82] Agus D. B., Gambhir S. S., Pardridge W. M. (1997). Vitamin C crosses the blood-brain barrier in the oxidized form through the glucose transporters. *Journal of Clinical Investigation*.

[83] Sotiriou S., Gispert S., Cheng J. (2002). Ascorbic-acid transporter Slc23a1 is essential for vitamin C transport into the brain and for perinatal survival. *Nature Medicine*.

[84] Spector R., Greenwald L. L. (1978). Transport and metabolism of vitamin B6 in rabbit brain and choroid plexus. *The Journal of Biological Chemistry*.

[85] Liang W. J., Johnson D., Jarvis S. M. (2001). Vitamin C transport systems of mammalian cells. *Molecular Membrane Biology*.

[86] Reis H. J., Guatimosim C., Paquet M. (2009). Neuro-transmitters in the central nervous system & their implication in learning and memory processes. *Current Medicinal Chemistry*.

[87] Geldenhuys W. J., Allen D. D. (2012). The blood-brain barrier choline transporter. *Central Nervous System Agents in Medicinal Chemistry*.

[88] King M., Su W., Chang A., Zuckerman A., Pasternak G. W. (2001). Transport of opioids from the brain to the periphery by P-glycoprotein: peripheral actions of central drugs. *Nature Neuroscience*.

[89] Zheng G., Zhang Z., Lockman P. R. (2010). Bis-azaaromatic quaternary ammonium salts as ligands for the blood-brain barrier choline transporter. *Bioorganic and Medicinal Chemistry Letters*.

[90] Mittapalli R. K., Manda V. K., Adkins C. E., Geldenhuys W. J., Lockman P. R. (2010). Exploiting nutrient transporters at the blood-brain barrier to improve brain distribution of small molecules. *Therapeutic Delivery*.

[91] Young R. K., Villalobos A. R. (2014). Stress-induced stimulation of choline transport in cultured choroid plexus epithelium exposed to low concentrations of cadmium. *The American Journal of Physiology: Regulatory, Integrative and Comparative Physiology*.

[92] Comley R. A., Salinas C. A., Slifstein M. (2013). Monoamine transporter occupancy of a novel triple reuptake inhibitor in baboons and humans using positron emission tomography. *Journal of Pharmacology and Experimental Therapeutics*.

[93] Mark C., Bornatowicz B., Mitterhauser M. (2013). Development and automation of a novel NET-PET tracer: [^11^C]Me@APPI. *Nuclear Medicine and Biology*.

[94] Daws L. C. (2009). Unfaithful neurotransmitter transporters: focus on serotonin uptake and implications for antidepressant efficacy. *Pharmacology and Therapeutics*.

[95] Eisenhofer G. (2001). The role of neuronal and extraneuronal plasma membrane transporters in the inactivation of peripheral catecholamines. *Pharmacology & Therapeutics*.

[96] Duan H., Wang J. (2010). Selective transport of monoamine neurotransmitters by human plasma membrane monoamine transporter and organic cation transporter 3. *Journal of Pharmacology and Experimental Therapeutics*.

[97] Kristensen A. S., Andersen J., Jørgensen T. N. (2011). SLC6 neurotransm it ter transporters: structure, function, and regulation. *Pharmacological Reviews*.

[98] Vornicescu C., Boşca B., Crişan D. (2013). Neuroprotective effect of melatonin in experimentally induced hypobaric hypoxia. *Romanian Journal of Morphology and Embryology*.

[99] Campos L. A., Cipolla-Neto J., Michelini L. C. (2013). Melatonin modulates baroreflex control via area postrema. *Brain and Behavior*.

[100] Dobolyi A., Kékesi K. A., Juhász G., Székely A. D., Lovas G., Kovács Z. (2014). Receptors of peptides as therapeutic targets in epilepsy research. *Current Medicinal Chemistry*.

[101] Cano V., Merino B., Ezquerra L., Somoza B., Ruiz-Gayo M. (2008). A cholecystokinin-1 receptor agonist (CCK-8) mediates increased permeability of brain barriers to leptin. *British Journal of Pharmacology*.

[102] Lo C. C., Langhans W., Georgievsky M. (2012). Apolipoprotein AIV requires cholecystokinin and vagal nerves to suppress food intake. *Endocrinology*.

[103] Hoyda T. D., Smith P. M., Ferguson A. V. (2009). Gastrointestinal hormone actions in the central regulation of energy metabolism: potential sensory roles for the circumventricular organs. *International Journal of Obesity*.

[104] McGonigle P. (2011). Peptide therapeutics for CNS indications. *Biochemical Pharmacology*.

[105] Mazza M., Notman R., Anwar J. (2013). Nanofiber-based delivery of therapeutic peptides to the brain. *ACS Nano*.

[106] Wong D. L., Tai T. C., Wong-Faull D. C. (2012). Epinephrine: a short- and long-term regulator of stress and development of illness: a potential new role for epinephrine in stress. *Cellular and Molecular Neurobiology*.

[107] Johansson J., Landgren M., Fernell E. (2011). Altered tryptophan and alanine transport in fibroblasts from boys with attention-deficit/hyperactivity disorder (ADHD): an in vitro study. *Behavioral and Brain Functions*.

[108] Sozio P., Cerasa L. S., Laserra S. (2013). Memantine-sulfur containing antioxidant conjugates as potential prodrugs to improve the treatment of Alzheimer's disease. *European Journal of Pharmaceutical Sciences*.

[109] Hughes C. G., Patel M. B., Pandharipande P. P. (2012). Pathophysiology of acute brain dysfunction: what's the cause of all this confusion?. *Current Opinion in Critical Care*.

[110] Patrick R. P., Ames B. N. (2014). Vitamin D hormone regulates serotonin synthesis. Part 1: relevance for autism. *The FASEB Journal*.

[111] Chern C. M., Liao J. F., Wang Y. H., Shen Y. C. (2012). Melatonin ameliorates neural function by promoting endogenous neurogenesis through the MT2 melatonin receptor in ischemic-stroke mice. *Free Radical Biology and Medicine*.

[112] Molnár G., Faragó N., Kocsis A. K. (2014). GABAergic neurogliaform cells represent local sources of insulin in the cerebral cortex. *The Journal of Neuroscience*.

[113] Nelson L., Tabet N., Richardson C., Gard P. (2013). Antihypertensives, angiotensin, glucose and Alzheimer's disease. *Expert Review of Neurotherapeutics*.

[114] Rodriguez P. L., Jiang S., Fu Y., Avraham S., Avraham H. K. (2014). The proinflammatory peptide substance P promotes blood-brain barrier breaching by breast cancer cells through changes in microvascular endothelial cell tight junctions. *International Journal of Cancer*.

[115] Catravas J. D., Gillis C. N. (1983). Single-pass removal of [14C]-5-hydroxytryptamine and [3H]norepinephrine by rabbit lung, *in vivo*: kinetics and sites of removal. *Journal of Pharmacology and Experimental Therapeutics*.

[116] Chemuturi N. V., Donovan M. D. (2007). Role of organic cation transporters in dopamine uptake across olfactory and nasal respiratory tissues. *Molecular Pharmaceutics*.

[117] Eisenhofer G. (2001). The role of neuronal and extraneuronal plasma membrane transporters in the inactivation of peripheral catecholamines. *Pharmacology and Therapeutics*.

[118] Ramamoorthy S., Prasad P. D., Kulanthaivel P., Leibach F. H., Blakely R. D., Ganapathy V. (1993). Expression of a cocaine-sensitive norepinephrine transporter in the human placental syncytiotrophoblast. *Biochemistry*.

[119] Sánchez del Pino M. M., Hawkins R. A., Peterson D. R. (1995). Biochemical discrimination between luminal and abluminal enzyme and transport activities of the blood-brain barrier. *Journal of Biological Chemistry*.

[120] Betz A. L. (1986). Transport of ions across the blood-brain barrier. *Federation Proceedings*.

[121] Nag S. (1990). Ultracytochemical localisation of Na+,K+-ATPase in cerebral endothelium in acute hypertension. *Acta Neuropathologica*.

[122] Mir L. M., Banoun H., Paoletti C. (1988). Introduction of definite amounts of nonpermeant molecules into living cells after electropermeabilization: direct assess to the cytosol. *Experimental Cell Research*.

[123] Pardridge W. M. (1983). Brain metabolism: a perspective from the blood-brain barrier. *Physiological Reviews*.

[124] Smith Q. R., Momma S., Aoyagi M., Rapoport S. I. (1987). Kinetics of neutral amino acid transport across the blood-brain barrier. *Journal of Neurochemistry*.

[125] Kayakabe M., Kakizaki T., Kaneko R. (2014). Motor dysfunction in cerebellar Purkinje cell-specific vesicular GABA transporter knockout mice. *Frontiers in Cellular Neuroscience*.

[126] Betz A. L., Goldstein G. W. (1978). Polarity of the blood-brain barrier: neutral amino acid transport into isolated brain capillaries. *Science*.

[127] Shank R. P., Campbell LeM. G. (1982). Glutamine and alpha-ketoglutarate uptake and metabolism by nerve terminal enriched material from mouse cerebellum. *Neurochemical Research*.

[128] Divito C. B., Underhill S. M. (2014). Excitatory amino acid transporters: roles in glutamatergic neurotransmission. *Neurochemistry International*.

[129] Hayashi K., Yamamoto S., Ohe K., Miyoshi A., Kawasaki T. (1980). Na^+^-gradient-dependent transport of l-proline and analysis of its carrier system in brush-border membrane vesicles of the guinea-pig ileum. *Biochimica et Biophysica Acta*.

[130] Bradbury M. W. B. (1985). The blood-brain barrier: transport across the cerebral endothelium. *Circulation Research*.

[131] Kuo Y., Gitschier J., Packman S. (1997). Developmental expression of the mouse mottled and toxic milk genes suggests distinct functions for the Menkes and Wilson disease copper transporters. *Human Molecular Genetics*.

[132] Vanduyn N., Settivari R., Levora J., Zhou S., Unrine J., Nass R. (2013). The metal transporter SMF-3/DMT-1 mediates aluminum-induced dopamine neuron degeneration. *Journal of Neurochemistry*.

[133] Bressler J. P. (2007). Metal transporters in intestine and brain: their involvement in metal-associated neurotoxicities. *Human & Experimental Toxicology*.

[134] Garrick M. D., Dolan K. G., Horbinski C. (2003). DMT1: a mammalian transporter for multiple metals. *BioMetals*.

[135] Shawki A., Knight P. B., Maliken B. D., Niespodzany E. J., MacKenzie B. (2012). H^+^-coupled divalent metal-ion transporter-1: functional properties, physiological roles and therapeutics. *Current Topics in Membranes*.

[136] Solute carrier family 11 (proton-coupled divalent metal ion transporters), member 2.

[137] Vidal S., Belouchi A. M., Cellier M., Beatty B., Gros P. (1995). Cloning and characterization of a second human NRAMP gene on chromosome 12q13. *Mammalian Genome*.

[138] Au C., Benedetto A., Aschner M. (2008). Manganese transport in eukaryotes: the role of DMT1. *Neurotoxicology*.

[139] Gunshin H., Mackenzie B., Berger U. V. (1997). Cloning and characterization of a mammalian proton-coupled metal-ion transporter. *Nature*.

[140] Aschner M. (2006). The transport of manganese across the bloodbrain barrier. *Neurotoxicology*.

[141] Ke Y., Chang Y. Z., Duan X. L. (2006). Age-dependent and iron-independent expression of two mRNA isoforms of divalent metal transporter 1 in rat brain. *Neurobiology of Aging*.

[142] Jamieson S. E., White J. K., Howson J. M. M. (2005). Candidate gene association study of solute carrier family 11a members 1 (*SLC11A1*) and 2 (*SLC11A2*) genes in Alzheimer's disease. *Neuroscience Letters*.

[143] Blasco H., Vourc'h P., Nadjar Y. (2011). Association between divalent metal transport 1 encoding gene (SLC11A2) and disease duration in amyotrophic lateral sclerosis. *Journal of the Neurological Sciences*.

[144] He Q., Du T., Yu X. (2011). DMT1 polymorphism and risk of Parkinson's disease. *Neuroscience Letters*.

[145] Xiong L., Dion P., Montplaisir J. (2007). Molecular genetic studies of *DMT1* on 12q in French-Canadian restless legs syndrome patients and families. *American Journal of Medical Genetics B: Neuropsychiatric Genetics*.

[146] Yao D., Mackenzie B., Ming H. (2000). A novel system A isoform mediating Na^+^/neutral amino acid cotransport. *Journal of Biological Chemistry*.

[147] van Winkle L. J., Iannaccone P. M., Campione A. L., Garton R. L. (1990). Transport of cationic and zwitterionic amino acids in preimplantation at conceptuses. *Developmental Biology*.

[148] Kerper L. E., Ballatori N., Clarkson T. W. (1992). Methylmercury transport across the blood-brain barrier by an amino acid carrier. *The American Journal of Physiology—Regulatory Integrative and Comparative Physiology*.

[149] Hughes C. C. W., Lantos P. L. (1989). Uptake of leucine and alanine by cultured cerebral capillary endothelial cells. *Brain Research*.

[150] Wiley D. T., Webster P., Gale A., Davis M. E. (2013). Transcytosis and brain uptake of transferrin-containing nanoparticles by tuning avidity to transferrin receptor. *Proceedings of the National Academy of Sciences of the United States of America*.

[151] Kreuter J. (2014). Drug delivery to the central nervous system by polymeric nanoparticles: what do we know?. *Advanced Drug Delivery Reviews*.

[152] Xiao G., Gan L. S. (2013). Receptor-mediated endocytosis and brain delivery of therapeutic biologics. *International Journal of Cell Biology*.

[153] Kamalinia G., Khodagholi F., Atyabi F. (2013). Enhanced brain delivery of deferasirox-lactoferrin conjugates for iron chelation therapy in neurodegenerative disorders: in vitro and in vivo studies. *Mol Pharm*.

[154] Manich G., Cabezón I., del Valle J. (2013). Study of the transcytosis of an anti-transferrin receptor antibody with a Fab′ cargo across the blood-brain barrier in mice. *European Journal of Pharmaceutical Sciences*.

[155] Tsuji A., Tamai I. I. (1999). Carrier-mediated or specialized transport of drugs across the blood-brain barrier. *Advanced Drug Delivery Reviews*.

[156] Simionescu M., Ghitescu L., Fixman A., Simionescu N. (1987). How plasma macromolecules cross the endothelium. *News in Phsyiological Sciences*.

[157] Pardridge W. M., Kang Y. S., Yang J., Buciak J. L. (1995). Enhanced cellular uptake and in vivo biodistribution of a monoclonal antibody following cationization. *Journal of Pharmaceutical Sciences*.

[158] Fishman J. B., Rubin J. B., Handrahan J. V., Connor J. R., Fine R. E. (1987). Receptor-mediated transcytosis of transferrin across the blood-brain barrier. *Journal of Neuroscience Research*.

[159] Duffy K. R., Pardridge W. M. (1987). Blood-brain barrier transcytosis of insulin in developing rabbits. *Brain Research*.

[160] Banks W. A., Kastin A. J. (1996). Reversible association of the cytokines MIP-1α and MIP-1*β* with the endothelia of the blood-brain barrier. *Neuroscience Letters*.

[161] Duffy K. R., Pardridge W. M., Rosenfeld R. G. (1988). Human blood-brain barrier insulin-like growth factor receptor. *Metabolism: Clinical and Experimental*.

[162] Moore M. S., Mahaffey D. T., Brodsky F. M., Anderson R. G. (1987). Assembly of clathrin-coated pits onto purified plasma membranes. *Science*.

[163] Stahl P., Schwartz A. L. (1986). Receptor-mediated endocytosis. *The Journal of Clinical Investigation*.

[164] Gonatas N. K., Stieber A., Hickey W. F., Herbert S. H. (1984). Endosomes and Golgi vesicles in adsorptive and fluid phase endocytosis. *Journal of Cell Biology*.

[165] Pardridge W. M. (1999). Vector-mediated drug delivery to the brain. *Advanced Drug Delivery Reviews*.

[166] Cordon-Cardo C., O'Brien J. P., Casals D. (1989). Multidrug-resistance gene (P-glycoprotein) is expressed by endothelial cells at blood-brain barrier sites. *Proceedings of the National Academy of Sciences of the United States of America*.

[167] Kusuhara H., Suzuki H., Sugiyama Y. (1998). The role of P-glycoprotein and canalicular multispecific organic anion transporter in the hepatobiliary excretion of drugs. *Journal of Pharmaceutical Sciences*.

[168] Tamai I., Ogihara T., Takanaga H., Maeda H., Tsuji A. (2000). Anion antiport mechanism is involved in transport of lactic acid across intestinal epithelial brush-border membrane. *Biochimica et Biophysica Acta: Biomembranes*.

[169] Banks W. A., Kastin A. J., Maness L. M., Huang W., Jaspan J. B. (1995). Permeability of the blood-brain barrier to amylin. *Life Sciences*.

[170] Bian-Sheng J., Cen J., Liu L., He L. (2013). *In vitro* and *in vivo* study of dodichyl phosphate on the efflux of P-glycoprotein at the blood brain. *International Journal of Developmental Neuroscience*.

[171] Tamai I., Tsuji A. (1996). Drug delivery through the blood-brain barrier. *Advanced Drug Delivery Reviews*.

[172] Seelig A., Gottschlich R., Devant R. M. (1994). A method to determine the ability of drugs to diffuse through the blood- brain barrier. *Proceedings of the National Academy of Sciences of the United States of America*.

[173] Ningaraj N. S., Rao M. K., Black K. L. (2003). Adenosine 5′-triphosphate-sensitive potassium channel-mediated blood-brain tumor barrier permeability increase in a rat Brain tumor model. *Cancer Research*.

[174] Ningaraj N. S., Rao M., Hashizume K., Asotra K., Black K. L. (2002). Regulation of blood-brain tumor barrier permeability by calcium-activated potassium channels. *Journal of Pharmacology and Experimental Therapeutics*.

[175] Rautio J., Chikhale P. J. (2004). Drug delivery systems for brain tumor therapy. *Current Pharmaceutical Design*.

[176] Etame A. B., Diaz R. J., Smith C. A., Mainprize T. G., Hynynen K., Rutka J. T. (2012). Focused ultrasound disruption of the blood-brain barrier: a new frontier for therapeutic delivery in molecular neurooncology. *Neurosurgical Focus*.

[177] Liu H.-L., Yang H.-W., Hua M.-Y., Wei K.-C. (2012). Enhanced therapeutic agent delivery through magnetic resonance imaging-monitored focused ultrasound blood-brain barrier disruption for brain tumor treatment: an overview of the current preclinical status. *Neurosurgical Focus*.

[178] Blakeley J. (2008). Drug delivery to brain tumors. *Current Neurology and Neuroscience Reports*.

[179] Black K. L., Ningaraj N. S. (2004). Modulation of brain tumor capillaries for enhanced drug delivery selectively to brain tumor. *Cancer Control*.

[180] Fross R. D., Warnke P. C., Groothuis D. R. (1991). Blood flow and blood-to-tissue transport in 9L gliosarcomas: the role of the brain tumor model in drug delivery research. *Journal of Neuro-Oncology*.

[181] Weyerbrock A., Walbridge S., Saavedra J. E., Keefer L. K., Oldfield E. H. (2011). Differential effects of nitric oxide on blood-brain barrier integrity and cerebral blood flow in intracerebral C6 gliomas. *Neuro-Oncology*.

[182] Upadhyay R. K. (2014). Drug delivery systems, CNS protection and the blood brain barrier. *BioMed Research International*.

